# Cryogenic Gas-Phase Infrared Ion Spectroscopy of Ultraviolet-Induced Nucleotide Photoproducts

**DOI:** 10.1021/acs.analchem.5c05815

**Published:** 2025-11-21

**Authors:** Gurpur Rakesh D. Prabhu, Michael Götze, Kim Greis, América Y. Torres-Boy, Marc Safferthal, Dominika Strzelecka, Carla Kirschbaum, Nimish D. Deshpande, Niklas Geue, Gerard Meijer, Gert von Helden, Kevin Pagel

**Affiliations:** a Department of Biology, Chemistry, Pharmacy, 9166 Freie Universität Berlin, Altensteinstraße 23a, Berlin 14195, Germany; b Department of Molecular Physics, Fritz Haber Institute of the Max Planck Society, Faradayweg 4-6, Berlin 14195, Germany

## Abstract

Ultraviolet (UV) exposure induces cross-linked pyrimidine dimers in nucleic acids, primarily forming cyclobutane pyrimidine dimers and 6–4 pyrimidine–pyrimidone adducts. These photoproducts exist in multiple isomeric forms, and various dimeric combinations involving thymine, cytosine, and uracil have been documented since the 1960s. Mass spectrometry (MS) has been pivotal in identifying these species, although condensed-phase spectroscopy remains essential for full structural elucidation. This study integrates MS with gas-phase infrared (IR) spectroscopy to obtain vibrational spectra (800–1900 cm^–1^) of UV-induced photoproducts from mono- and dinucleotides. Following nanoelectrospray ionization and in-source collision-induced dissociation, fragment ions—commonly used in tandem MS experiments to identify the photoproducts—are embedded in superfluid helium clusters at 0.37 K to measure high-resolution IR action spectra. These spectra are then compared with density functional theory-calculated spectra of various candidate isomers to facilitate structural assignment without reference standards. This combined approach enables detailed characterization of complex, low-abundance biomolecules beyond the reach of conventional MS.

## Graphical abstract

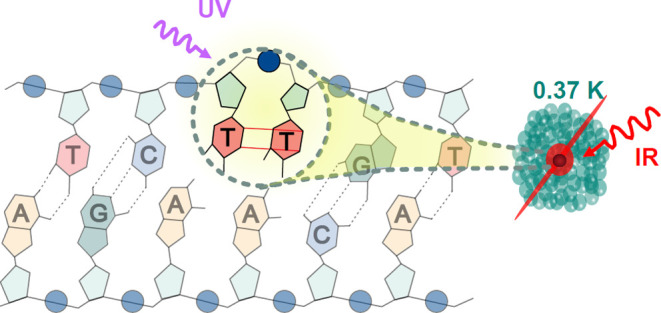

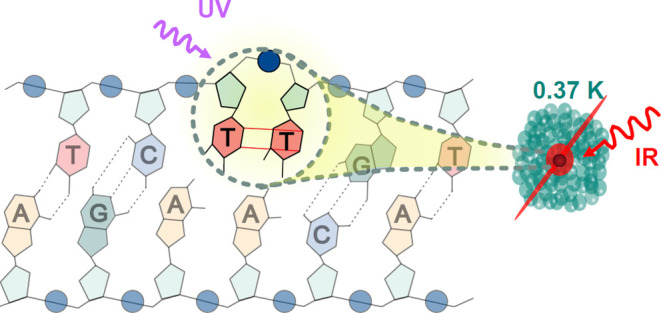

## Introduction

In the 19th century, ultraviolet (UV) light was discovered to induce photochemical reactions^1^ and damage living systems.^2,3^ Subsequent studies linked these effects to nucleic acid damage by comparing bactericidal action spectra^4^ with the UV absorption profiles of nucleobases^5,6^ —insights made possible through advances in UV spectroscopy.^7^ Upon UV exposure, nucleic acids form cross-linked photoproducts between adjacent or nonadjacent pyrimidines (Figure 1),^8–11^ disrupting genetic function. Remarkably, nucleobases dissipate excitation energy via internal conversion in under a picosecond,^12^ a process thought to have contributed to their evolutionary selection as genetic carriers.^13–15^ In addition, to counteract UV damage, organisms employ repair enzymes like photolyases^16–18^ and nucleotide excision repair systems.^19^ Depending on damage severity, cells may also activate protective mechanisms to prevent mutation transmission.^20–22^ In humans, defects in these pathways are linked to diseases marked by increased UV sensitivity.^23–25^

**Figure 1:**
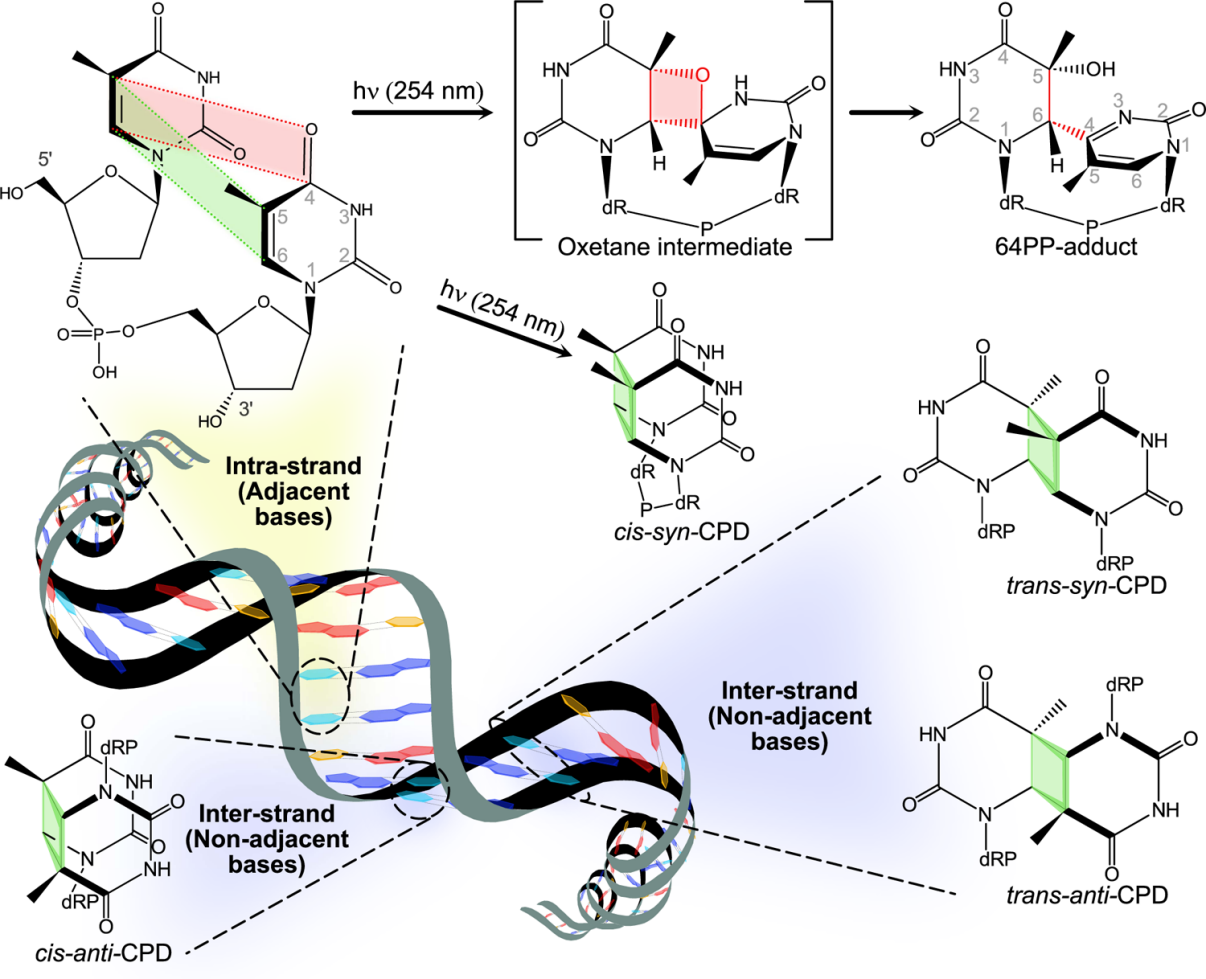
Photochemical reaction scheme showing the formation of different isomers of dimeric thymidine photoproducts in DNA depending on the orientation and position of nucleotides. CPD – cyclobutane pyrimidine dimer; dRP – deoxyribose phosphate.

UV irradiation of nucleic acids at 254 nm produces two major classes of isomeric photoproducts: cyclobutane pyrimidine dimers (CPDs) and 6–4 pyrimidine-pyrimidone (64PP) adducts (Figure 1).^11^ These can form among thymine, cytosine, and uracil bases—including both homodimers and cross-reactive species—with thymine dimers being most prevalent.^26–28^ The formation of CPD diastereomers (*cis-syn*, *cis-anti*, *trans-syn*, *trans-anti*) and 64PP-adducts depends on nucleobase position and orientation within strands.^10,29^ Upon irradiation to 320 nm UV light, 64PP-adduct can convert into its Dewar valence isomer. CPDs arise via [2 + 2] photocycloaddition between adjacent C5 = C6 double bonds,^27,30,31^ while 64PP-adduct likely forms through a similar reaction involving the C5 = C6 bond of the 5′ base and the C4 carbonyl of the 3′ base via an oxetane intermediate.^32,33^ These photoproducts have been observed in UV-irradiated mononucleotides,^9^ dinucleotides,^8^ oligonucleotides,^34^ DNA,^10,35^ RNA,^36,37^ and human skin.^38^ Additionally, a unique lesion—5-thyminyl-5,6-dihydrothymine—forms via radical pathways in dehydrated bacterial spore DNA.^10,39–41^

Mass spectrometry (MS), a sensitive gas-phase technique, has been instrumental in identifying cross-linked photoproducts since the 1960s.^42–44^ Structural elucidation, however, has relied heavily on complementary condensed-phase spectroscopic methods, often following liquid chromatography (LC) purification.^45–48^ Electrospray ionization (ESI)^49,50^ enabled the direct coupling of LC to MS, facilitating real-time analysis of LC eluates. While LC-MS/MS offers high sensitivity for detecting fragment ions of photoproducts in mono-^9^ and dinucleotide^8^ and intact nucleic acids,^35,36,38^ it lacks comprehensive structural resolution—especially for isomeric species. Ion mobility spectrometry (IMS) combined with MS/MS has successfully separated CPD isomers (*cis-syn* and *trans-anti*) and measured their collision cross sections (CCS),^51^ though full resolution of complex isomeric mixtures remains unreported. This goal may be achievable using advanced IMS platforms with superior resolving capabilities.^52,53^ Nevertheless, CCS measurements offer limited structural resolution as they provide only a rotationally averaged cross-section of an ion that interacts with a buffer gas. This study employs a technique that integrates the sensitivity of MS with gas-phase infrared (IR) ion spectroscopy to elucidate the structures of photoproducts by probing their fragment ions. The findings reveal that this combined approach enables precise structural characterization of complex, low-abundance biomolecules that conventional MS alone cannot resolve.

## Experimental Section

### Preparation of Photoproducts

Solutions of thymidine monophosphate (TMP), dithymidine monophosphate (TpT), and uridine monophosphate (UMP) were individually irradiated with UV light at 254 nm to generate cross-linked photoproducts (TMP_XL_, TpT_XL_, and UMP_XL_), which were subsequently purified by size-exclusion chromatography (SEC). Each purified sample was then analyzed using nano-ESI tandem MS (timsTOF Pro, Bruker, Bremen, Germany) in positive-ion mode (nano-ESI capillary voltage: 1.0 kV). Photoproduct formation was confirmed by monitoring fragment ions (*m*/*z* 449 for TMP_XL_ and TpT_XL_, as well as *m*/*z* 437 for UMP_XL_) generated by collision induced dissociation (CID) with collision-gas nitrogen at different collision voltages (5 to 40 V). LC–CID-IMS-MS experiments were performed using a Synapt G2-S mass spectrometer (Waters Corporation, MA, USA) equipped with traveling wave IMS and an Acquity UPLC system. Further experimental details on synthesis, purification, and LC-IMS separation of photoproducts are provided in the Supporting Information.

### Cryogenic Gas-Phase IR Ion Spectroscopy

A custom-built instrument was used for cryogenic gas-phase IR ion spectroscopy; a detailed description of the instrument can be found elsewhere.^54^ Briefly, photoproducts ionized using nano-ESI (∼ 1 kV) are fragmented by in-source CID. The fragment ions are then *m*/*z*-selected using a quadrupole mass analyzer and directed toward a cold (90 K) hexapole ion trap for accumulation and thermalization. Simultaneously, superfluid helium clusters (10^4^ to 10^6^ atoms) are generated using a pulsed Even-Lavie valve by supersonic expansion of helium gas into vacuum of the mass spectrometer. The thermalized ions stored in the cold trap are picked up by helium clusters traversing the trap at a speed of about 500 m s^–1^ and are carried forward into the interaction region by overcoming the trapping potential barrier due to their kinetic energy. The embedded ions are rapidly cooled to subkelvin temperatures (0.37 K) by the helium environment. The helium clusters containing ions are guided along a pathway that overlaps with the tunable IR free-electron laser (FEL) beamline at the Fritz Haber Institute (Berlin).^55^ In this study, the FEL was operated in the mid-IR region (800–1900 cm^–1^), delivering IR radiation as ∼ 10 μs macro-pulses at a 10 Hz repetition rate, each composed of ∼ 10 ps micropulses occurring at 1 GHz.

Helium atoms exhibit minimal interaction with the embedded ion and remain transparent to IR radiation. Upon laser irradiation, the ion absorbs resonant photons, resulting in vibrational excitation. The energy of the excited ion is rapidly dissipated into the helium matrix through intramolecular vibrational energy redistribution, leading to the evaporation of some surrounding helium atoms. This process cools the ion back to subkelvin temperatures and returns it to its vibrational ground state. Following repeated cycles of photon absorption and energy transfer, bare unsolvated ions are ultimately detected via time-of-flight mass spectrometry. The ion signal recorded as a function of the tunable laser wavelength yields an IR spectrum. Unlike room-temperature gas-phase IR spectroscopy, here, cooling the ions thermally depopulates the number of quantum states. Furthermore, since excitation is performed from the vibrational ground state during the several cycles of energy absorption and release, spectral congestion is reduced resulting in a highly resolved spectrum that can be readily compared with computer simulated spectra for molecular structure elucidation.

## Theoretical Methods

Initial structures of isomeric candidates were digitally created on GaussView software (version 6.0.16). Protonation sites and conformational spaces of the nucleotide photoproduct fragment ions of each isomer were explored using CREST software (version 2.9)^56^ with the semiempirical method GFN2-xTB.^57^ Among the generated conformer ensemble, structures of around 20 lowest energy conformers (positively charged, singlet spin state; see Table S1 for energetics) were geometry-optimized at PBE0/6–31G­(d)^58,59^ level of theory using Gaussian (version 16) with empirical dispersion (GD3BJ).^60^ Among the geometry-optimized structures, those with relative electronic energies (ΔE) lower than 15 kJ mol^–1^ were reoptimized at a higher level of theory PBE0/6–311+G­(d,p) with empirical dispersion (GD3BJ), and their harmonic frequencies were computed. In addition, electrostatic charges within the molecule were computed using the Merz–Singh–Kollman scheme,^61^ with the additional command for reproducing the overall molecular dipole moment, as implemented in Gaussian using the keyword pop = (mk,dipole). All harmonic frequencies were scaled by an empirical factor of 0.965.^62,63^ Energies specified in this study for conformers of various isomers are relative to the lowest energy conformer; ΔE values (in kJ mol^–1^) correspond to the sum of electronic and zero-point vibrational energies. Computed CCS values of conformers in nitrogen at 298.15 K were generated using the trajectory method implemented in the HPCCS software.^64^

## Results and Discussion

### Tandem Mass Spectrometry

A common approach for differentiating isobaric species in MS involves monitoring their fragmentation patterns. The gas-phase dimers of nucleotides (TMP and UMP) and their corresponding cross-linked photoproducts are isobaric species (see Figure S1). Likewise, TpT and its cross-linked derivative TpT_XL_ share identical masses. Nevertheless, tandem MS fragmentation of nucleotides in positive ion mode, before and after UV irradiation, clearly demonstrate that the ions at *m*/*z* 449 for TMP_XL_ and TpT_XL_, and *m*/*z* 437 for UMP_XL_, originate from cross-linked photoproducts (Figure 2 and Figure S1). However, unambiguous identification of different isomeric photoproducts based on fragmentation patterns can be challenging, as distinct isomers may generate identical fragmentation profiles (Figure S2). In negative ion mode, the formation of corresponding fragment ions requires significantly higher collision voltages (Figure S3), which may not be readily achievable via in-source CID implemented in this study. Therefore, ions generated in the positive ion mode were selected for IR spectroscopy to identify the photoproducts.

**Figure 2:**
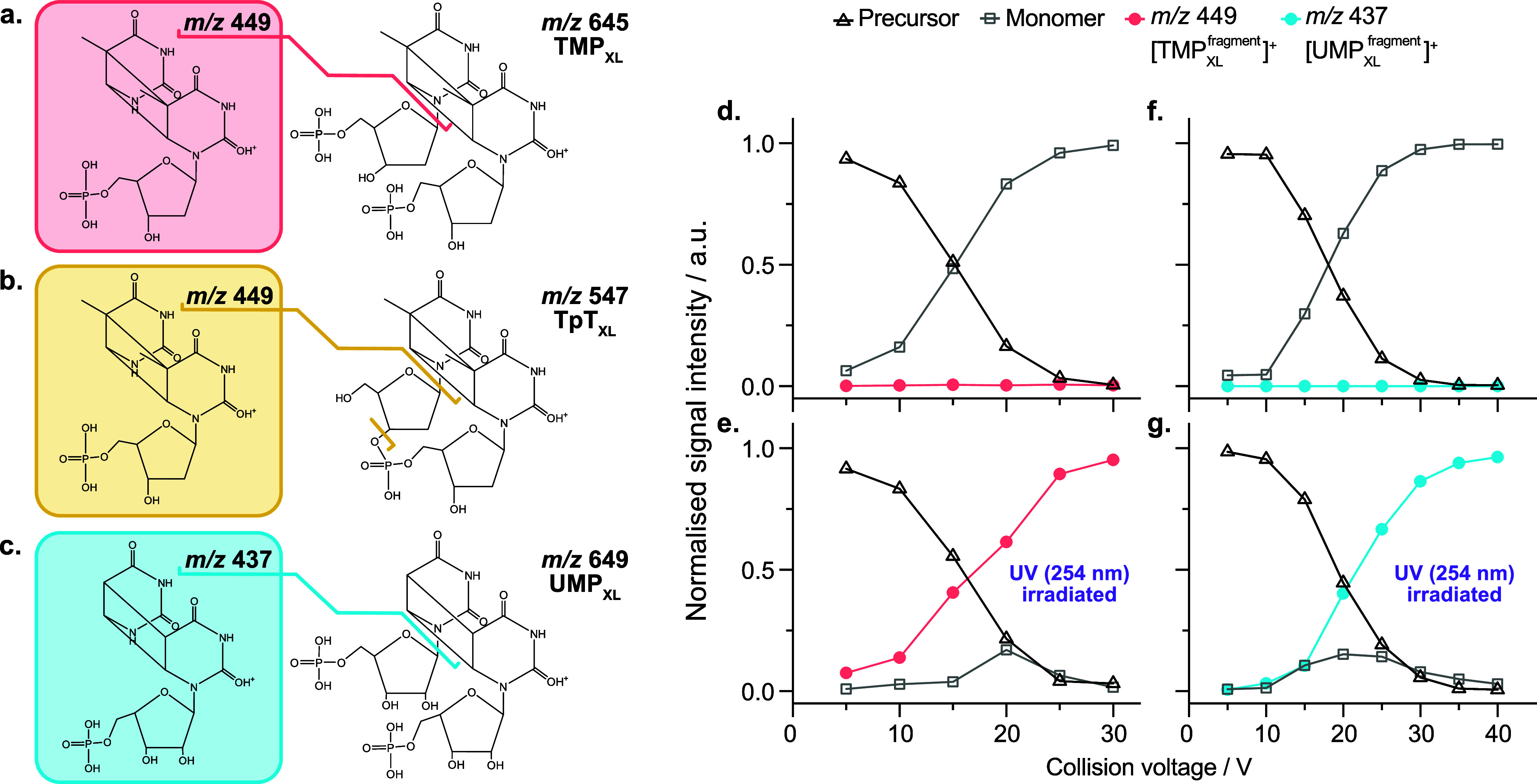
Tandem MS analysis of nucleotide photoproducts. Tentative structures of fragment and precursor ions: (a) TMP_XL_; (b) TpT_XL_; (c) UMP_XL_. Of the five isomers depicted in Figure 1, only the *cis-syn*-CPD isomer is illustrated here. Fragmentation yield at different collision voltages before and after UV irradiation: (d: before, e: after) TMP_XL_; and (f: before, g: after) UMP_XL_. See Figure S1 for mass spectra.

The presence of fragments corresponding to the *m*/*z* of mononucleotides in the spectra obtained after UV irradiation can be due to incomplete separation of unreacted nucleotides from photoproducts by SEC, resulting from a low reaction yield (<5%). To assess the stability of the fragments retaining cross-links in TMP_XL_ and UMP_XL_ relative to their respective gas-phase dimers of mononucleotides, collision voltage scans were conducted both before and after UV irradiation. The persistence of cross-links at a collision voltage of 30 V (Figure 2) highlights the structural stability of these cross-linked species. To prevent unreacted mononucleotides—present as gas-phase dimers—from contributing to the IR spectrum, fragment ions retaining the cross-links were selected for gas-phase IR spectroscopic analysis (Figure S4). This approach leverages the ability of gas-phase IR ion spectroscopy to provide structural information on the probed ion, whether it is a precursor or a fragment. Analyzing fragment ions reduces the system size for quantum chemical calculations, as they contain fewer atoms than the precursor ions. Fragment ions also produce less complex spectra, simplifying the analysis.

### Liquid Chromatography–Ion Mobility Mass Spectrometry

UV irradiation of mono- and dinucleotide solutions likely results in multiple photoproducts due to random diffusional encounters between nucleotides adopting diverse spatial orientations.^65^ The LC-MS/MS fragment ion chromatograms (Figure 3 and Figure S5) clearly indicate the presence of multiple peaks corresponding to fragment ions at *m*/*z* 449 and *m*/*z* 437. The chromatogram of TMP_XL_ exhibits a dominant peak (i) alongside a smaller peak (ii). For TpT_XL_, the chromatogram reveals three prominent peaks (i–iii) and two minor ones (iv and v). For UMP_XL_, the chromatogram displays two prominent peaks (i and ii), along with a poorly resolved minor feature positioned between them. Since HILIC separation is governed by analyte structure—particularly polarity and hydrophilic partitioning with the stationary phase—variations in the number, orientation, and positioning of hydroxyl groups among analytes lead to differential interactions with the stationary phase, thereby facilitating their separation. The observed chromatographic peaks can therefore be attributed to distinct photoproducts.

**Figure 3:**
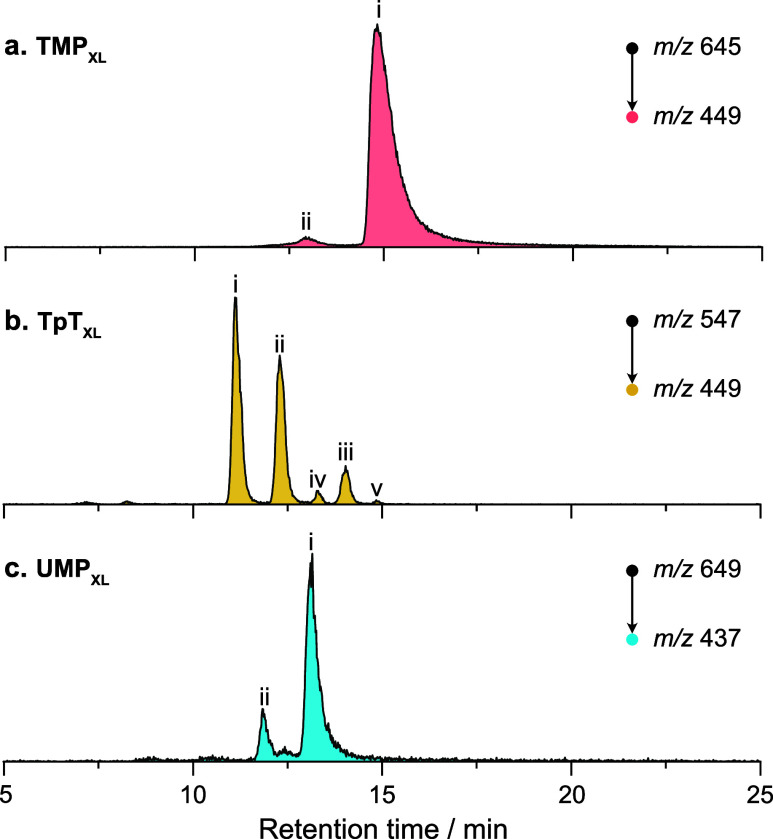
LC-MS/MS extracted ion chromatograms of CID fragments of photoproducts. (a) TMP_XL_; (b) TpT_XL_; (c) UMP_XL_. Peaks are ranked based on quantities of analytes. See Figure S5 for chromatograms of precursor ions.

While analyte identification via retention time matching with reference standards is achievable, it poses challenges when dealing with these photoproducts lacking such standards. Likewise, analyte separation by IMS^51,66^ may also prove limited when using IMS platforms with low resolving power, particularly in cases where arrival time distributions of isomers overlap (Figures S5–S7) and their predicted CCS values differ only marginally (Table S2), often within the calculation error of the algorithms.^64^ This is evident especially in the case of TpT_XL_ fragment ion at *m*/*z* 449 (Figure S6c) and precursor ion at *m*/*z* 547 (Figure S6d), for which a significant overlap is seen in the arrival time distributions for structures ii to v that are in fact baseline separated by LC. In the case of the TMP_XL_-fragment as well (Figure S6a), the presence of structure ii may be easily overlooked due to its overshadowing by the more dominant presence of structure i in the mobilogram.

## Cryogenic Gas-Phase IR Ion Spectroscopy

Unlike the low-resolution structural information obtained from IMS, gas-phase IR spectroscopy provides detailed structural insights by probing molecular vibrations, which are unique to different molecular structures. By acquiring vibrational spectra of gas-phase ions and comparing them with simulated spectra, structural assignments can be made. Originally developed in the 1970s,^67^ the technique has evolved significantly^68^ and can be performed at room temperature,^69,70^ at cryogenic temperatures via messenger tagging (10–60 K),^71–73^ or within superfluid helium clusters (0.37 K),^54^ on mass-selected and/or mobility-separated ions.^74^ It has been successfully applied to diverse molecular classes, including glycans,^75^ peptides,^76^ nucleotides,^62,63,77–79^ lipids,^80^ proteins,^81^ polyaromatic hydrocarbons,^82^ reactive intermediates,^83,84^ and tandem MS fragment ions of metabolites.^85^ Furthermore, UV-IR double-resonance gas-phase spectroscopy can be used to record conformer- or isomer-selective vibrational spectra of electronically excited ions.^86–88^

The experimental IR spectrum of the TMP_XL_ fragment ion at *m*/*z* 449 (Figure 4) reveals seven vibrational bands in the region between 1600 and 1800 cm^–1^ — a strong band centered around 1690 cm^–1^, and three weaker bands on either side. In the spectrum recorded at a laser macro-pulse energy of ∼ 30 mJ, the intense band appeared as a broad, irregular feature. To investigate the possible presence of overlapping transitions within this region, the spectrum was reacquired at a reduced macro-pulse energy of approximately 15 mJ, under which the weaker bands exhibited diminished intensities. The strong presence of the band centered around 1690 cm^–1^ suggests that it may originate from the structure corresponding to the dominant peak in the LC chromatogram.

**Figure 4:**
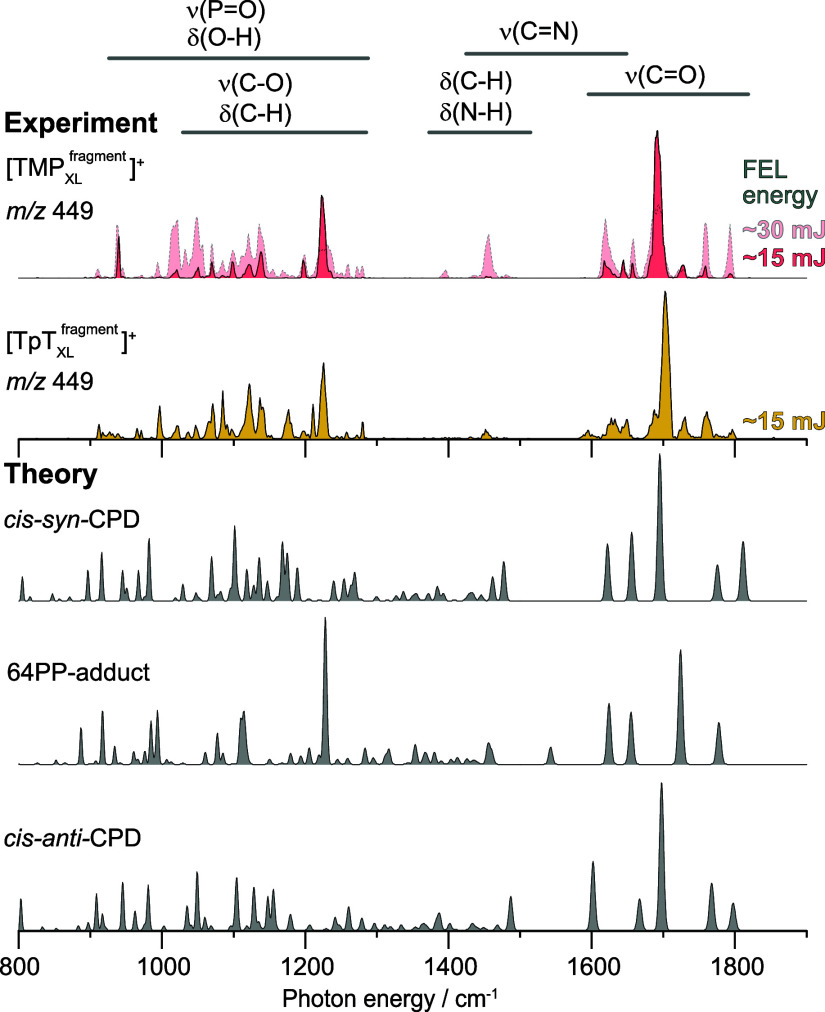
Comparison of experimental cryogenic gas-phase IR spectra of TMP_XL_ and TpT_XL_ fragment ions at *m*/*z* 449 with DFT-predicted vibrational transitions for various photoproducts. The observed spectral features for TMP_XL_ are consistent with predicted transitions of the *cis-syn*-CPD and 64PP-adduct, while those for TpT_XL_ additionally align with the *cis-anti*-CPD isomer.

Interestingly, the IR spectrum of the TpT_XL_ fragment ion at *m*/*z* 449 exhibits a strong band centered around 1700 cm^–1^, accompanied by a subtle shoulder band of lower intensity near 1690 cm^–1^. While the three weaker bands on either side of the strong band are consistent with those observed for TMP_XL_, an additional weak band is also present at 1595 cm^–1^. For both TMP_XL_ and TpT_XL_, the region between 1300 and 1590 cm^–1^ is predominantly baseline, featuring only a weak band at 1450 cm^–1^ and several very weak transitions scattered between 1350 and 1470 cm^–1^. Lastly, the region from 900 to 1300 cm^–1^ displays several bands for both samples, with only one prominent band centered at approximately 1225 cm^–1^. The presence of additional spectral features in the TpT_XL_ spectrum aligns with its LC chromatogram, which features peaks corresponding to three major and two minor photoproduct isomers. The presence of two adjacent thymine bases within a TpT molecule likely contributes to the formation of a higher number of photoproducts compared to TMP, where photoproducts form only through interactions between thymine bases on separate molecules.

To elucidate the structures of fragment ions of TMP_XL_ and TpT_XL_ at *m*/*z* 449, computer-simulated spectra were calculated for five nucleotide photoproduct isomeric candidates—namely, four CPD diastereoisomers and one 64PP-adduct—reported previously in the literature.^8–11,34,8–11,42–44^ Note that previously reported spore photoproduct and Dewar valence isomer of the 64PP-adduct were excluded, as these photoproducts are unlikely to form under the synthetic conditions employed in this study.^9,10^ It is also important to note that, for each isomer, the fragment ion at *m*/*z* 449 for TMP_XL_ can result from the cleavage of a sugar–phosphate group linked to either of the thymine bases. Similarly, the fragment ion at *m*/*z* 449 for TpT_XL_ may arise from cleavage of a sugar group linked to either thymine base. Calculations were limited to a single type of fragment ion to minimize computational costs. The experimental IR spectra were carefully compared with simulated spectra of multiple conformers of the five isomers, all within a ΔE of 15 kJ mol^–1^. Figure 4 presents one of the best-matching simulated spectra selected from the conformers of each isomer (see Figures S8–S12).

The strong band centered around 1690 cm^–1^ in the TMP_XL_ experimental spectrum aligns well with the predicted C = O stretching transition in the simulated spectrum of the lowest-energy conformer of the *cis-syn*-CPD isomer (Figure 4 and Figure S8). Two weaker transitions on either side of the strong band roughly correspond to features in the simulated spectrum— specifically, bands at 1656 cm^–1^, 1759 cm^–1^, and 1795 cm^–1^ are attributed to C = O stretching, while the band at 1617 cm^–1^ corresponds to N–C–N antisymmetric stretching. The weak bands observed near 1644 cm^–1^ and 1725 cm^–1^ in the experimental spectrum does not correspond to any predicted transition from the *cis-syn*-CPD conformers. Notably, a conformer of the 64PP-adduct exhibits a predicted strong C = O transition in this region. Additionally, the prominent band around 1225 cm^–1^ in the experimental spectrum, which lacks a theoretical counterpart among the *cis-syn*-CPD conformers, aligns with a predicted transition for P = O stretching in the simulated spectrum of the same 64PP-adduct conformer, suggesting a possible structural contribution to the recorded spectrum (Figure S9). Although the ΔE of this conformer is 5.8 kJ mol^–1^, its presence is conceivable at 90 K trap temperature. The weak band near 1644 cm^–1^ corresponds to a theoretical C = O stretching mode of the 64PP-adduct (Figure S9). The simulated spectra of both *cis-syn*-CPD and the 64PP-adduct predict weak bands scattered between 1400 cm^–1^ and 1500 cm^–1^, which are also observed in the recorded spectrum.

The spectral region spanning 800–1200 cm^–1^ is less diagnostic compared to the C = O stretching domain; however, the observed features generally align with predictions for this range. The experimental bands that match predicted transitions of either the *cis-syn*-CPD or 64PP-adduct are consistent with the observation that the LC chromatogram of TMP_XL_ features two peaks (Figure 3a). The fact that 64PP-adduct generally exhibits a lower quantum yield than CPD isomers^26,89^ suggests that the dominant peak in the chromatogram may be attributed to the *cis-syn*-CPD, while the minor peak likely corresponds to the 64PP-adduct.

In the case of TpT_XL_, the presence of a strong band centered around 1700 cm^–1^, along with a weaker shoulder at 1690 cm^–1^ (where TMP_XL_ exhibits a strong band) and a weak band at 1595 cm^–1^, suggests the existence of additional photoproducts beyond the *cis-syn*-CPD and 64PP-adduct. Notably, the lowest-energy conformer of *cis-anti*-CPD exhibits a predicted strong C = O transition near 1700 cm^–1^. Additionally, its predicted spectrum includes a weak transition corresponding to the feature at 1595 cm^–1^. Interestingly, conformers of *cis-anti*-CPD isomer with ΔE 5.3 kJ mol^–1^, 5.5 kJ mol^–1^, and 7.5 kJ mol^–1^, also exhibit similar spectrum (Figure S10). The simulated spectra of the CPD isomers—*trans-syn* and *trans-anti*—contain some transitions that match the experimental data; however, prominent bands around 1675 cm^–1^ and 1680 cm^–1^, respectively, are not observed in the recorded spectrum. Accordingly, the three major peaks observed in the LC chromatogram of TpT_XL_ (Figure 3b) can be attributed to the *cis-syn*-CPD, *cis-anti*-CPD, and 64PP-adduct isomers. The structures associated with the minor chromatographic peaks (iv and v) are presumably present in quantities insufficient to yield detectable contributions to the IR spectrum.

The *cis-anti*-CPD isomer is observed exclusively in TpT, but not in TMP. Moreover, high-mass ions at *m*/*z* 1093 ([2TpT_XL_ + H]^+^) and *m*/*z* 1115 ([2TpT_XL_ + Na]^+^) with signal intensities equivalent to the monomeric species—*m*/*z* 547 ([TpT_XL_ + H]^+^) and *m*/*z* 569 ([TpT_XL_ + Na]^+^), respectively—are detected in the mass spectrum of TpT_XL_, whereas TMP_XL_ shows no such signals (Figure S13). Furthermore, fragmentation of the peak at *m*/*z* 1093 also yields the fragment ion at *m*/*z* 449. Since in-source-CID was used to generate fragment ions from nonselected precursor ions for IR spectroscopy, it is likely that the ions at *m*/*z* 449—filtered by a quadrupole and stored in a cold hexapole ion trap—include fragments originating from precursor ions at *m*/*z* 547 ([TpT_XL_ + H]^+^) and *m*/*z* 1093 ([2TpT_XL_ + H]^+^). The formation of the *cis-anti*-CPD isomer in TpT_XL_ may, therefore, result from intermolecular cross-linking rather than intramolecular cross-linking between adjacent thymine bases in TpT. Since the recorded IR spectral features of the ion at *m*/*z* 449 align with simulated spectra for *cis-syn*-CPD, *cis-anti*-CPD, and 64PP-adduct structures, it is important to note that tandem MS analysis alone cannot reliably identify photoproducts in positive ion mode.

In addition to photoproducts formed from deoxyribonucleotides, those generated in the ribonucleotide uridine monophosphate (UMP) are also equally relevant. The experimental IR spectrum of the UMP_XL_ fragment ion at *m*/*z* 437 (Figure 5) recorded at a laser macro-pulse energy of ∼ 15 mJ reveals several bands between 1600 and 1810 cm^–1^. A prominent band appears at 1685 cm^–1^, accompanied by a band of approximately three-fourths its intensity at 1700 cm^–1^, as well as weaker features at 1617, 1630, 1650, 1660, 1769, and 1804 cm^–1^. At a laser macro-pulse energy of approximately 30 mJ, the recorded spectrum failed to resolve the prominent bands, instead displaying a broad and irregular feature between 1640 and 1720 cm^–1^ (Figure S14). Nonetheless, a weak band near 1400 cm^–1^ is discernible. Within the 1250–1600 cm^–1^ region, only three spectral features are observed: a sharp band at 1481 cm^–1^, and two broad bands centered around 1400 and 1450 cm^–1^. As observed for TMP_XL_ and TpT_XL_, the spectral region below 1250 cm^–1^ exhibits several features, some of which remain imperfectly resolved even in the spectrum recorded at low laser macro-pulse energy. Theoretical spectra for UMP_XL_ fragment ion at *m*/*z* 437 were calculated for five structural candidates—four CPD diastereomers^90,91^ and a 64PP-adduct^92^—as these have been reported previously. The experimental IR spectra were compared with simulated spectra of multiple conformers corresponding to the isomers, each within a ΔE threshold of 15 kJ mol^–1^. Figure 5 showcases simulated spectra with close agreement to the experimental data, selected from conformers of each isomer (see Figures S14–S18).

**Figure 5:**
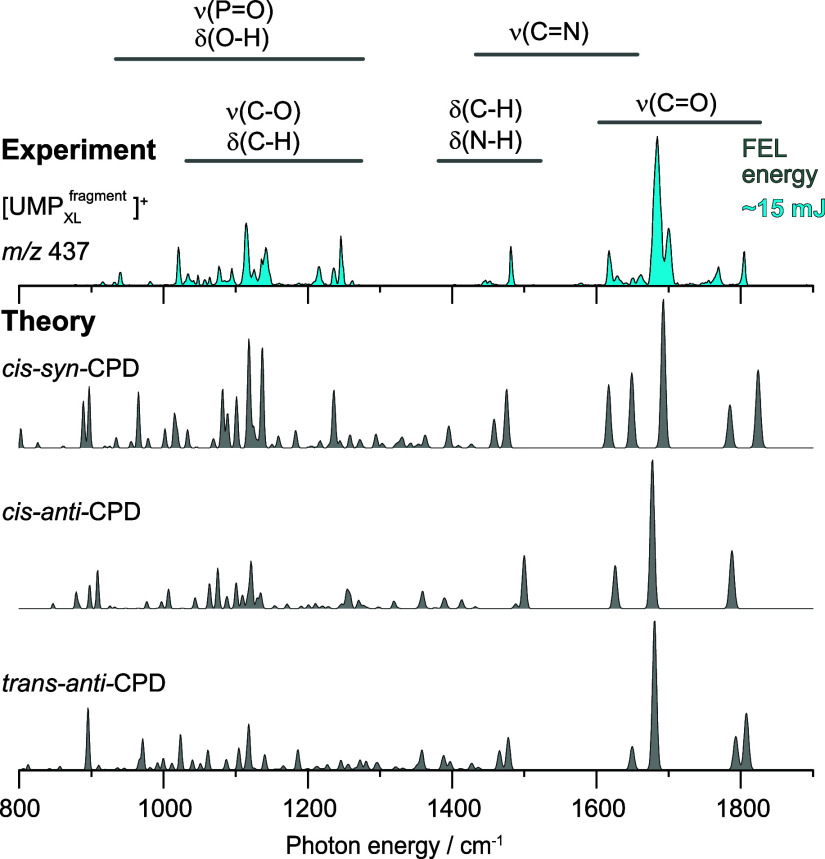
Comparison of experimental cryogenic gas-phase IR spectra of UMP_XL_ fragment ion at *m*/*z* 437 with DFT-predicted transitions for different photoproducts. Given the inherent limitations of DFT methods, definitive structural assignment is challenging, and the observed spectrum may correspond to any of these three isomers.

The transitions predicted in the simulated spectrum for a conformer of the *cis-syn*-CPD isomer with ΔE = 5.3 kJ mol^–1^ closely match the experimentally observed features, except for slight shifts in two bands at the far right of the spectrum and a diminished intensity of the band at 1650 cm^–1^. Moreover, the most prominent bands in the C = O stretching region—1685 cm^–1^ and 1700 cm^–1^—observed in the experimental spectrum suggest the presence of an additional photoproduct. It is likely that the intense band at 1685 cm^–1^ arises from the strongest predicted transition of the *cis-anti*-CPD conformer with ΔE = 3.5 kJ mol^–1^, while the band at 1700 cm^–1^ may correspond to the strongest predicted transition of the *cis-syn*-CPD isomer. Interestingly, some of the predicted transitions for the *trans-anti*-CPD isomer also match the experimental data—particularly the strongest band and two minor features near 1475 and 1650 cm^–1^. Nevertheless, theory predicts two bands near 1800 cm^–1^ for *trans-anti*-CPD isomer, whereas only one is observed experimentally. Considering the two prominent peaks with an unresolved feature positioned between them, as observed in the LC chromatogram of UMP_XL_ (Figure 3c), the *cis-syn*-CPD and *cis-anti*-CPD isomers are presumably the major photoproducts for UMP that form the fragment ion at *m*/*z* 437, although the presence of trace amounts of the *trans-anti*-CPD isomer cannot be entirely ruled out. In contrast, the presence of *trans-syn*-CPD can be excluded, as its most prominent predicted transitions are not experimentally observed. As the predicted transitions for the 64PP-adduct do not match the experimental spectrum, it is certain that the 64PP-adduct does not yield the fragment ion at *m*/*z* 437. Nevertheless, it is possible that the loss of a water molecule from the 64PP-adduct at the 5′-end thymine results in an ion at *m*/*z* 419, rather than *m*/*z* 437 (Figure S2).^9,58^

## Conclusion

Cryogenic gas-phase IR ion spectroscopy was employed to investigate the UV-induced photoproducts of TMP, TpT, and UMP for molecular structure determination. Although MS offers high sensitivity and selectivity and enables analysis with minimal sample volumes, the vibrational signatures obtained from gas-phase ions yield structural insights that are beyond the reach of conventional MS techniques. Structural elucidation of multiple photoproducts was achieved without reference standards by comparing high-resolution experimental vibrational spectra with simulated spectra. For TMP_XL_, the fragment ion at *m*/*z* 449 arises from both the *cis-syn*-CPD and 64PP adducts, whereas for TpT_XL_, the *cis-anti*-CPD isomer can also yield the same ion. In the case of UMP_XL_, the fragment ion at *m*/*z* 437 originates predominantly from *cis-syn*-CPD and *cis-anti*-CPD isomers.

Isomeric photoproducts commonly arise upon UV irradiation of mono- or dinucleotide solutions, and their presence is further validated by LC-based separation evidence. Additionally, the fragment ions analyzed here are routinely employed in LC-MS/MS-based identification of photoproducts at TpT sites, following enzymatic digestion of nucleic acids to release these modified regions. The complex mixture of isomeric photoproducts and limited sample quantities impedes structural elucidation using condensed-phase spectroscopy. The gas-phase IR spectroscopy technique utilized here is primarily useful for determining the structure of a probed ion; whether it is a precursor or a fragment. It offers a distinct advantage over condensed-phase techniques when only limited sample quantities are available, as is often the case with UV-induced photoproducts. Although there are reports on the use of the room-temperature variant of the technique, often coupled with LC separation, for analyzing other biologically relevant isomeric small molecules, such as metabolites,^93–95^ our study is the first to apply cryogenic gas-phase IR spectroscopy for the direct analysis of a nucleotide photoproduct isomeric mixture. Due to limited accessibility of the specialized, custom-built instruments—such as the one employed in this study—the full potential of this technique remains underexplored. Gas-phase separation of the isomers using IMS prior to spectroscopic analysis can reduce the complexity of the analysis.^96,97^ Efforts are currently underway to develop an instrument that enables cryogenic messenger-tagging spectroscopy of mass-selected and/or mobility-separated ions. Additionally, it would also be interesting to perform UV-IR double-resonance gas-phase spectroscopy on nucleotides. According to DFT calculations, the OH-stretching region (3000–3800 cm^–1^) also exhibits diagnostic bands; however, multiple isomers and conformers have predicted bands at similar frequencies, which may complicate the analysis. Nevertheless, it would be valuable for laboratories equipped with tunable tabletop lasers to measure the 3000–3800 cm^–1^ region to identify the photoproducts.

## Supplementary Material



## Notes

The Supporting Information is available free of charge on the ACS Publications Web site. The Supporting Information is available free of charge at https://pubs.acs.org/doi/10.1021/acs.analchem.5c05815.

Additional experimental details; additional figures (Figures S1–S18); additional tables (Tables S1 and S2); atomic XYZ coordinates of optimized structures of the conformers. (PDF)
