# Supplementary material for: Cryogenic Gas-Phase Infrared Ion Spectroscopy of Ultraviolet-Induced Nucleotide Photoproducts
Source: Anal Chem. 2025 Nov 21;97(48):26868–76. doi: 10.1021/acs.analchem.5c05815 (PMC12874228; doi:10.1021/acs.analchem.5c05815)
Supplement: Supplementary file 1 [file ac5c05815_si_001.pdf]

Supporting Information

Cryogenic gas-phase infrared ion spectroscopy of ultraviolet-induced nucleotide photoproducts

Gurpur Rakesh D. Prabhu,[ab] Michael Götze,[ab] Kim Greis,[ab] América Y. Torres-Boy,[b] Marc Safferthal,[ab] Dominika Strzelecka,[ab] Carla Kirschbaum,[ab] Nimish D. Deshpande,[a] Niklas Geue,[ab] Gerard Meijer,[b] Gert von Helden,[b] and Kevin Pagel[ab]*

[a] *Department of Biology, Chemistry, Pharmacy, Freie Universität Berlin, Altensteinstraße 23a, 14195 Berlin, Germany*

[b] *Department of Molecular Physics, Fritz Haber Institute of the Max Planck Society, Faradayweg 4-6, 14195 Berlin, Germany*

Table of Contents

ADDITIONAL EXPERIMENTAL DETAILS 3
Materials.......... 3
Synthesis of nucleotide photoproducts 3
Preparation of nano-electrospray emitters.......... 3
Liquid Chromatography – Ion Mobility Spectrometry – Tandem Mass Spectrometry.......... 3
Drift-tube Ion Mobility-Mass Spectrometry 4
Table S1. Energetics and theoretical CCS of conformers of photoproduct isomers. 5
Figure S1. Tandem MS spectra of nucleotides before and after UV (254 nm) irradiation 10
Figure S2. Tentative structural assignments of nucleotide fragments generated by tandem MS 11
Figure S3. Tandem MS of nucleotide photoproducts in positive and negative ion modes..........12
Figure S4. Mass spectra of in-source CID fragment ions of photoproducts 13
Figure S5. LC-MS/MS chromatograms of the photoproduct mixtures 14
Figure S6. LC-MS/MS-TWIMS mobilograms of photoproduct fragment ions and precursor ions 15
Figure S7. Drift-tube ion mobility-mass spectrometry of fragment ions of photoproducts..........16
Figure S8. Comparison of experimental IR spectra of TMP_{XL} and TpT_{XL} fragment ions at *m/z* 449 with DFT calculated spectra of conformers of *cis-syn*-CPD isomer. 17
Figure S9. Comparison of experimental IR spectra of TMP_{XL} and TpT_{XL} fragment ions at *m/z* 449 with DFT calculated spectra of conformers of 64PP-adduct isomer. 18
Figure S10. Comparison of experimental IR spectra of TMP_{XL} and TpT_{XL} fragment ions at *m/z* 449 with DFT calculated spectra of conformers of *cis-anti*-CPD isomer 19
Figure S11. Comparison of experimental IR spectra of TMP_{XL} and TpT_{XL} fragment ions at *m/z* 449 with DFT calculated spectra of conformers of *trans-syn*-CPD isomer..........20
Figure S12. Comparison of experimental IR spectra of TMP_{XL} and TpT_{XL} fragment ions at *m/z* 449 with DFT calculated spectra of conformers of *trans-anti*-CPD isomer..........21
Figure S13. Mass spectra of nucleotides after UV (254 nm) irradiation and putative mechanism of formation of *cis-anti*-CPD isomer upon UV irradiation of TpT solution. 22
Figure S14. Comparison of experimental IR spectrum of UMP_{XL} fragment ion at *m/z* 437 with DFT calculated spectra of conformers of *cis-syn*-CPD isomer..........23
Figure S15. Comparison of experimental IR spectrum of UMP_{XL} fragment ion at *m/z* 437 with DFT calculated spectra of conformers of *cis-anti*-CPD isomer 24
Figure S16. Comparison of experimental IR spectrum of UMP_{XL} fragment ion at *m/z* 437 with DFT calculated spectra of conformers of *trans-anti*-CPD isomer..........25
Figure S17. Comparison of experimental IR spectrum of UMP_{XL} fragment ion at *m/z* 437 with DFT calculated spectra of conformers of *trans-syn*-CPD isomer..........26
Figure S18. Comparison of experimental IR spectrum of UMP_{XL} fragment ion at *m/z* 437 with DFT calculated spectra of conformers of 64PP-adduct isomer..........27
Table S2. Comparison of experimental $^{DT}CCS_{N2}$ values with theoretical $^{TM}CCS_{N2}$ values 28
Atomic XYZ coordinates of optimized structures of the conformers 29
References:..........35

ADDITIONAL EXPERIMENTAL DETAILS

Materials

Thymidine monophosphate (TMP) and uridine monophosphate (UMP) were purchased from Sigma-Aldrich (Taufkirchen, Germany), and dithymidine monophosphate (TpT) was purchased from Biosynth (Bratislava, Slovakia). Methanol (LiChrosolv, hypergrade for LC-MS) and water (LiCrosolv, LC-MS grade) were purchased from Merck (Darmstadt, Germany). Acetonitrile was purchased from Honeywell (Morristown, USA). Ammonium acetate and ammonium formate were purchased from Merck (Darmstadt, Germany). Packing material (Biogel P-2) for size exclusion chromatography (SEC) was purchased from BioRad (Feldkirchen, Germany). All chemicals and solvents were used as received without further purification.

Synthesis of nucleotide photoproducts

Nucleotide standards were dissolved in water to prepare 10 mM, 1 mM, and 100 mM solutions of TMP, TpT, and UMP, respectively. All three solutions were then irradiated with UV light at 254 nm (~ 3 mW cm-2) for 4 hours using AnalytikJena's CX-2000 UV Cross-linker (Jena, Germany). Nucleotide solutions were placed on water-ice to improve the reaction yield. The three reaction mixtures were then lyophilized overnight and were subsequently re-dissolved in 100 mM ammonium acetate aqueous solutions. Unreacted nucleotides were separated from cross-linked photoproducts (TMP_{XL}, TpT_{XL}, and UMP_{XL}) by passing the solutions through a SEC column (packing material: Biogel P-2; mobile phase: 100 mM ammonium acetate aqueous solution; 160 cm x 1.5 cm, 300 mL bed volume). SEC fractions were lyophilized and were re-dissolved in 10 µL water and were stored as stock solutions at -25 °C. Working solutions were prepared by pipetting 1 µL of stock into 100 µL methanol:water (50:50; v/v).

Preparation of nano-electrospray emitters

For all the experiments using nano-electrospray for sample ionization, Borosilicate glass capillaries were pulled in-house to a tip with an inner diameter of < 3 µm using a micropipette puller (Model P-1000, Sutter Instrument, Novato, USA) and were coated with palladium-platinum alloy (80:20, w/w) by a sputter coater (Model 108auto, Cressington Scientific Instruments, Watford, UK). Tips were clipped with a tweezer and filled with 5-10 µL of sample for spraying.

Liquid Chromatography – Ion Mobility Spectrometry – Tandem Mass Spectrometry

LC-CID-IMS-MS experiments were performed using a Synapt G2-S mass spectrometer (Waters Corporation, MA, USA) equipped with travelling wave IMS and an Acquity UPLC system. Photoproducts were separated by hydrophilic interaction liquid chromatography (HILIC) using a 2.1 mm × 150 mm Acquity Premier BEH Amide column of 1.7 µm particle size (Waters, Manchester, UK). The column oven was set to 60 °C, and the flow rate was set to 0.4 mL min^{-1}. Solvent A was 5 mM ammonium formate in water and solvent B was acetonitrile. 2-5 µL of the sample solutions prepared in acetonitrile:water (90:10; v/v) were injected into the column.

TMP_{XL} was eluted using an isocratic gradient at 82% B, followed by linear gradient of 82-68% from 5 to 20 min. TpT_{XL} was eluted using an isocratic gradient at 90% B, followed by linear gradient of 90-78% from 5 to 20 min. UMP_{XL} was eluted using an isocratic gradient at 77% B, followed by linear gradient of 77-63% from 5 to 20 min. Eluates were ionized using ESI in positive-ion mode with a capillary voltage of 3.0 kV (LockSpray source, Waters). Other instrument parameters were: source temperature, 80 °C; desolvation temperature, 250 °C; cone gas (N_2) flow, 1 L h^{-1}; desolvation gas flow, 500 L h^{-1}; nebulizer gas pressure, 6 bar; and mass range, *m/z* 50 to *m/z* 1500. Fragmentation of the precursor ions (*m/z* 645 of TMP_{XL}; *m/z* 547 of TpT_{XL}; and *m/z* 649 of UMP_{XL}) was performed by CID (collision gas: argon) in the trap region with collision voltage set to 10 V. In particular, extracted ion chromatograms of fragment ions *m/z* 449 of TMP_{XL} and TpT_{XL}, as well as *m/z* 437 of UMP_{XL} were monitored to deduce the number of isomer populations.

Mobilograms of the fragment ions were recorded in the buffer gas N_2 using a travelling wave velocity of 1100 m s^{-1} with a wave height of 40 V. Helium cell and IMS gas flow were set to 180 mL min^{-1} and 90 mL min^{-1}, respectively. The transfer cell wave velocity was set to 220 m s^{-1} with a wave height of 4 V. Data were acquired and processed using MassLynx (version 2.0.7, Waters) and DriftScope (version 2.6, Waters) software.

Drift-tube Ion Mobility-Mass Spectrometry

Experimental CCS values were obtained using a customized Synapt G2-S HDMS ion mobility-mass spectrometer equipped with a drift tube,[1] replacing the standard traveling wave ion mobility cell.

Samples were ionized using nano-ESI in positive-ion mode (capillary voltage: ~1 kV) and directed into an argon-filled collision cell for fragmentation via CID, with the collision voltage set to 2 V. The fragment ions with *m/z* 449 (from TMP_{XL} and TpT_{XL}) and *m/z* 437 (from UMP_{XL}), generated from their respective precursor ions, were gently propelled through a nitrogen buffer gas (pressure: 1.8 torr) under the influence of a weak and uniform electric field applied across a 25.05 cm-long drift tube maintained at 25 °C to record their arrival time distributions. Arrival time distributions for the fragment ions were recorded at nine different drift voltages (V_D). Drift times corresponding to the peak maxima of unresolved arrival time distributions (see **Figure S7**), which exhibit minor contributions from two or more isomers (see **Figure S6**), were plotted against the inverse of V_D. Ion mobilities (K) were determined from the slope of the linear regression of drift time plotted against the inverse of V_D. The $^{DT}CCS_{N2}$ values were calculated using Mason-Schamp equation:[2]

$$K = \frac{3}{16}\frac{ze}{N}\sqrt{\frac{2\pi}{\mu k_B T}}\frac{1}{\Omega}$$

where, z is the charge of the analyte ion, e is elementary charge, N is the gas number density of the buffer gas, μ is the reduced mass of the analyte ion-buffer gas pair, k_B is Boltzmann constant, T is the buffer gas temperature, and Ω is the CCS of the analyte ion-buffer gas pair. K was transformed into the reduced ion mobility (K_0) before calculating $^{DT}CCS_{N2}$:

$$K_0 = K\frac{T_0}{T}\frac{p}{p_0}$$

Where, T_0 = 273.16 K (standard temperature), p_0 = 760 Torr (standard pressure), and p is recorded buffer gas pressure.

Owing to limited isomeric separation, the $^{DT}CCS_{N2}$ values obtained in this experiment allow only an approximate comparison between the size and shape of nucleotide photoproduct fragment ions and their predicted counterparts (see **Table S2**). This limitation is underscored by the LC-IMS-MS/MS data, which reveal the presence of multiple photoproducts capable of producing isobaric fragment ions.

Table S1. Energetics and theoretical CCS of conformers of photoproduct isomers. For each isomer, the structures of lowest energy conformers obtained from CREST were geometry optimized using Gaussian at PBE0/6-31G(d) with GD3BJ level of theory. Among the optimized structures, those with relative electronic energy (ΔE) < ~15 kJ mol^{-1} were reoptimized using Gaussian at a higher level of theory PBE0/6-311+G(d,p) with GD3BJ and their harmonic frequencies were computed. All ΔE values specified are sum of electronic and zero-point vibrational energies. Values highlighted in bold correspond to the conformers whose theoretical spectra are compared with experimental IR spectra (see **Figures S8-S12** and **S14-S18**) and theoretical $^{TM}CCS_{N2}$ values are calculated using HPCCS algorithm;[3] duplicate conformers and those with ΔE ≥15 kJ mol^{-1} are excluded. Rows highlighted with gray background correspond to the conformers for which the experimental IR spectra matches with theory.

***trans-syn*-CPD** isomer of **TMP_{XL}** and **TpT_{XL}** fragment ions at *m/z* 449			
CREST output: 34 conformers			
	ΔE (kJ mol^{-1})		$^{TM}CCS_{N2}$ (Å^2)
Conformer number	PBE0/ 6-31G(d) with GD3BJ	PBE0/ 6-311+G(d,p) with GD3BJ	
conf_0	8.0	**1.3**	193
conf_1	1.2	**3.2**	193
conf_2	17.1	**10.8**	191
conf_3	8.6	**2.3**	193
conf_4	27.3		
conf_5	0.0	**0.0**	191
conf_6	11.7	**1.9**	192
conf_7	13.2	**4.9**	196
conf_8	28.6		
conf_9	24.6		
conf_10	13.5	**4.0**	194
conf_11	26.6		
conf_12	26.2		
conf_13	31.0		
conf_14	4.2	**4.8**	193
conf_15	28.2		
conf_16	32.0		
conf_17	16.4	**6.8**	192
conf_18	27.2		
conf_19	20.4		

***trans-anti*-CPD** isomer of **TMP_{XL}** and **TpT_{XL}** fragment ions at *m/z* 449			
CREST output: 42 conformers			
	ΔE (kJ mol^{-1})		$^{TM}CCS_{N2}$ (Å^2)
Conformer number	PBE0/ 6-31G(d) with GD3BJ	PBE0/ 6-311+G(d,p) with GD3BJ	
conf_0	0.0	**0.2**	192
conf_1	1.9	**1.5**	192
conf_2	0.4	**0.0**	191
conf_3	0.4		
conf_4	11.3	**10.1**	193
conf_5	1.9	**1.3**	191
conf_6	1.9		
conf_7	30.4		
conf_8	18.7	21.2	
conf_9	9.4	**0.2**	193
conf_10	1.9		
conf_11	9.4		
conf_12	11.2	**8.7**	191
conf_13	16.3	17.6	
conf_14	7.6	**12.7**	193
conf_15	24.6		
conf_16	37.6		
conf_17	56.4		
conf_18	29.9		
conf_19	20.8		

Table S1 (continued). Energetics and theoretical CCS of conformers of photoproduct isomers.

***cis-syn*-CPD** isomer of **TMP**$_{XL}$ and **TpT**$_{XL}$ fragment ions at *m/z* 449

CREST output: 20 conformers

Conformer number	ΔE (kJ mol^{-1}) PBE0/ 6-31G(d) with GD3BJ	ΔE (kJ mol^{-1}) PBE0/ 6-311+G(d,p) with GD3BJ	$^{TM}CCS_{N2}$ (Å^2)
conf_0	0.0	**0.0**	186
conf_1	7.3	**8.5**	187
conf_2	11.3	**10.3**	189
conf_3	11.2		
conf_4	7.3		
conf_5	5.9	**5.6**	186
conf_6	39.6		
conf_7	31.9		
conf_8	44.2		
conf_9	28.2		
conf_10	22.5		
conf_11	31.9		
conf_12	16.5	**13.4**	188
conf_13	41.2		
conf_14	16.5		
conf_15	16.5		
conf_16	5.9		
conf_17	41.9		
conf_18	48.7		
conf_19	47.3		

***cis-anti*-CPD** isomer of **TMP**$_{XL}$ and **TpT**$_{XL}$ fragment ions at *m/z* 449

CREST output: 45 conformers

Conformer number	ΔE (kJ mol^{-1}) PBE0/ 6-31G(d) with GD3BJ	ΔE (kJ mol^{-1}) PBE0/ 6-311+G(d,p) with GD3BJ	$^{TM}CCS_{N2}$ (Å^2)
conf_0	4.5	**3.7**	195
conf_1	8.0	**6.1**	193
conf_2	7.2	6.1	
conf_3	17.7	21.9	
conf_4	4.5	3.6	
conf_5	4.5		
conf_6	4.5		
conf_7	17.0	3.7	
conf_8	2.2	**1.2**	193
conf_9	7.5	3.6	
conf_10	12.5	**14.4**	190
conf_11	4.5		
conf_12	19.9	18.3	
conf_13	8.3	**7.7**	193
conf_14	22.4		
conf_15	5.6	3.7	
conf_16	0.0	**0.0**	185
conf_17	7.1	**7.0**	190
conf_18	6.1	**7.5**	186
conf_19	9.3	**9.4**	193
conf_20	5.8	**5.8**	191
conf_21	10.1	**10.1**	192
conf_22	17.9	21.5	
conf_23	21.1		
conf_24	2.2		
conf_25	7.5	1.2	
conf_26	21.7		
conf_27	13.0	9.4	
conf_28	21.9		
conf_29	0.9	**5.3**	186
conf_30	1.3	**5.5**	186
conf_31	8.6	1.2	
conf_32	15.4	10.1	
conf_33	17.4	19.4	
conf_34	22.9		
conf_35	26.3		
conf_36	18.7	20.5	
conf_37	25.4		
conf_38	30.3		
conf_39	26.1		

Table S1 (continued). Energetics and theoretical CCS of conformers of photoproduct isomers.

64PP-adduct isomer of **TMP_{XL}** and **TpT_{XL}** fragment ions at *m/z* 449

CREST output: 23 conformers			
	ΔE (kJ mol^{-1})		
Conformer number	PBE0/ 6-31G(d) with GD3BJ	PBE0/ 6-311+G(d,p) with GD3BJ	$^{TM}CCS_{N2}$ (Å^2)
conf_0	4.8	**8.4**	193
conf_1	0.9	**2.6×10^{-3}**	194
conf_2	7.9	**5.8**	194
conf_3	0.9	**0.0**	194
conf_4	3.8	**5.9**	195
conf_5	3.4	**5.6**	195
conf_6	17.3		
conf_7	22.8		
conf_8	0.9		
conf_9	7.8	**8.1**	195
conf_10	15.6		
conf_11	0.2	**2.0**	195
conf_12	17.4		
conf_13	4.4×10^{-3}	**1.9**	195
conf_14	8.6×10^{-4}	1.9	
conf_15	11.1	**11.1**	194
conf_16	28.7		
conf_17	0.0	1.9	
conf_18	25.8		
conf_19	32.0		
conf_20	38.2		
conf_21	38.1		
conf_22	38.8		

64PP-adduct isomer of **UMP_{XL}** fragment ion at *m/z* 437

CREST output: 29 conformers			
	ΔE (kJ mol^{-1})		
Conformer number	PBE0/ 6-31G(d) with GD3BJ	PBE0/ 6-311+G(d,p) with GD3BJ	$^{TM}CCS_{N2}$ (Å^2)
conf_0	0.0	**0.0**	188
conf_1	6.3	**5.0**	189
conf_2	15.7	**13.9**	190
conf_3	16.4	15.5	
conf_4	21.3		
conf_5	9.3	**11.2**	188
conf_6	15.7		
conf_7	0.0		
conf_8	9.3		
conf_9	6.3		
conf_10	21.3		
conf_11	9.1	**11.6**	188
conf_12	8.8	**8.0**	189
conf_13	18.0	16.9	
conf_14	24.5		
conf_15	18.0		
conf_16	17.2	20.1	
conf_17	11.1	14.5	
conf_18	17.8	15.6	
conf_19	14.9	**13.2**	192

Table S1 (continued). Energetics and theoretical CCS of conformers of photoproduct isomers.

***cis-syn*-CPD** isomer of **UMP$_{XL}$** fragment ion at *m/z* 437				***cis-anti*-CPD** isomer of **UMP$_{XL}$** fragment ion at *m/z* 437			
CREST output: 27 conformers				CREST output: 31 conformers			
	ΔE (kJ mol^{-1})				ΔE (kJ mol^{-1})		
Conformer number	PBE0/ 6-31G(d) with GD3BJ	PBE0/ 6-311+G(d,p) with GD3BJ	$^{TM}CCS_{N2}$ (Å^2)	Conformer number	PBE0/ 6-31G(d) with GD3BJ	PBE0/ 6-311+G(d,p) with GD3BJ	$^{TM}CCS_{N2}$ (Å^2)
conf_0	0.0	**0.0**	184	conf_0	0.0	**0.0**	183
conf_1	5.5	**4.5**	185	conf_1	3.2	**3.5**	184
conf_2	15.8	**12.6**	185	conf_2	10.3	**8.8**	183
conf_3	5.5	4.5		conf_3	20.2		
conf_4	20.2			conf_4	19.3		
conf_5	10.9	**7.9**	184	conf_5	21.7		
conf_6	20.2			conf_6	19.5		
conf_7	20.2			conf_7	10.3		
conf_8	5.6	**5.3**	183	conf_8	21.7		
conf_9	18.6	14.9		conf_9	19.5		
conf_10	39.1			conf_10	11.6	**10.2**	183
conf_11	45.4			conf_11	20.7		
conf_12	18.6			conf_12	19.5		
conf_13	32.8			conf_13	14.0		
conf_14	44.2			conf_14	19.8		
conf_15	38.9			conf_15	12.5	**13.1**	187
conf_16	47.2			conf_16	19.6		
conf_17	30.7			conf_17	25.1		
conf_18	18.6			conf_18	11.0	**8.6**	183
conf_19	31.2			conf_19	28.1		

Table S1 (continued). Energetics and theoretical CCS of conformers of photoproduct isomers.

***trans-syn*-CPD** isomer of **UMP_{XL}** fragment ion at *m/z* 437

CREST output: 24 conformers			
	ΔE (kJ mol^{-1})		
Conformer number	PBE0/ 6-31G(d) with GD3BJ	PBE0/ 6-311+G(d,p) with GD3BJ	$^{TM}CCS_{N2}$ (Å^2)
conf_0	0.0	**0.0**	189
conf_1	21.6		
conf_2	3.3	**2.1**	187
conf_3	25.5		
conf_4	23.4		
conf_5	15.6	**9.8**	192
conf_6	26.5		
conf_7	34.9		
conf_8	26.8		
conf_9	40.3		
conf_10	14.7	**6.1**	191
conf_11	18.5	18.3	
conf_12	23.4		
conf_13	24.6		
conf_14	25.2		
conf_15	34.9		
conf_16	30.5		
conf_17	28.9		
conf_18	23.6		
conf_19	30.7		

***trans-anti*-CPD** isomer of **UMP_{XL}** fragment ion at *m/z* 437

CREST output: 16 conformers			
	ΔE (kJ mol^{-1})		
Conformer number	PBE0/ 6-31G(d) with GD3BJ	PBE0/ 6-311+G(d,p) with GD3BJ	$^{TM}CCS_{N2}$ (Å^2)
conf_0	12.6	**0.9**	191
conf_1	5.6	**4.2**	190
conf_2	4.8	**3.7**	190
conf_3	14.2	0.7	
conf_4	13.9	**0.0**	189
conf_5	16.0		
conf_6	16.5		
conf_7	6.2	**4.9**	189
conf_8	12.6		
conf_9	4.6	**1.8**	190
conf_10	24.7		
conf_11	4.6		
conf_12	0.0	**0.6**	188
conf_13	37.3		
conf_14	9.1	**10.7**	191
conf_15	14.6	19.3	

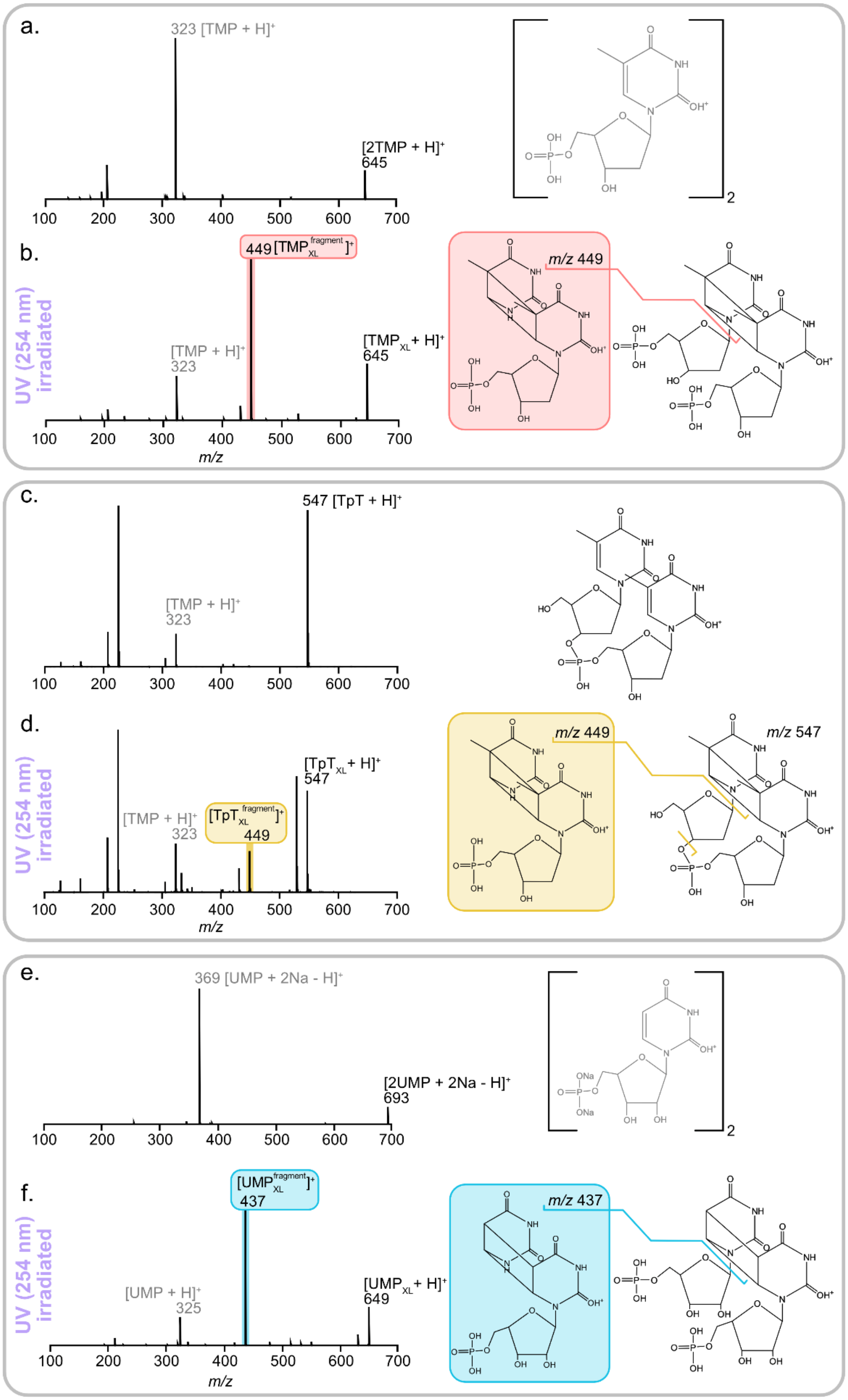

Figure S1. Tandem MS spectra of nucleotides before and after UV (254 nm) irradiation: (a) TMP; (b) TMP_{XL}; (c) TpT; (d) TpT_{XL}; (e) UMP; and (f) UMP_{XL}. Of the five isomers depicted in **Figure 1**, only the *cis-syn* isomer is illustrated here. Experiments were performed on a timsTOF Pro mass spectrometer (Bruker, Bremen, Germany) with collision gas nitrogen.

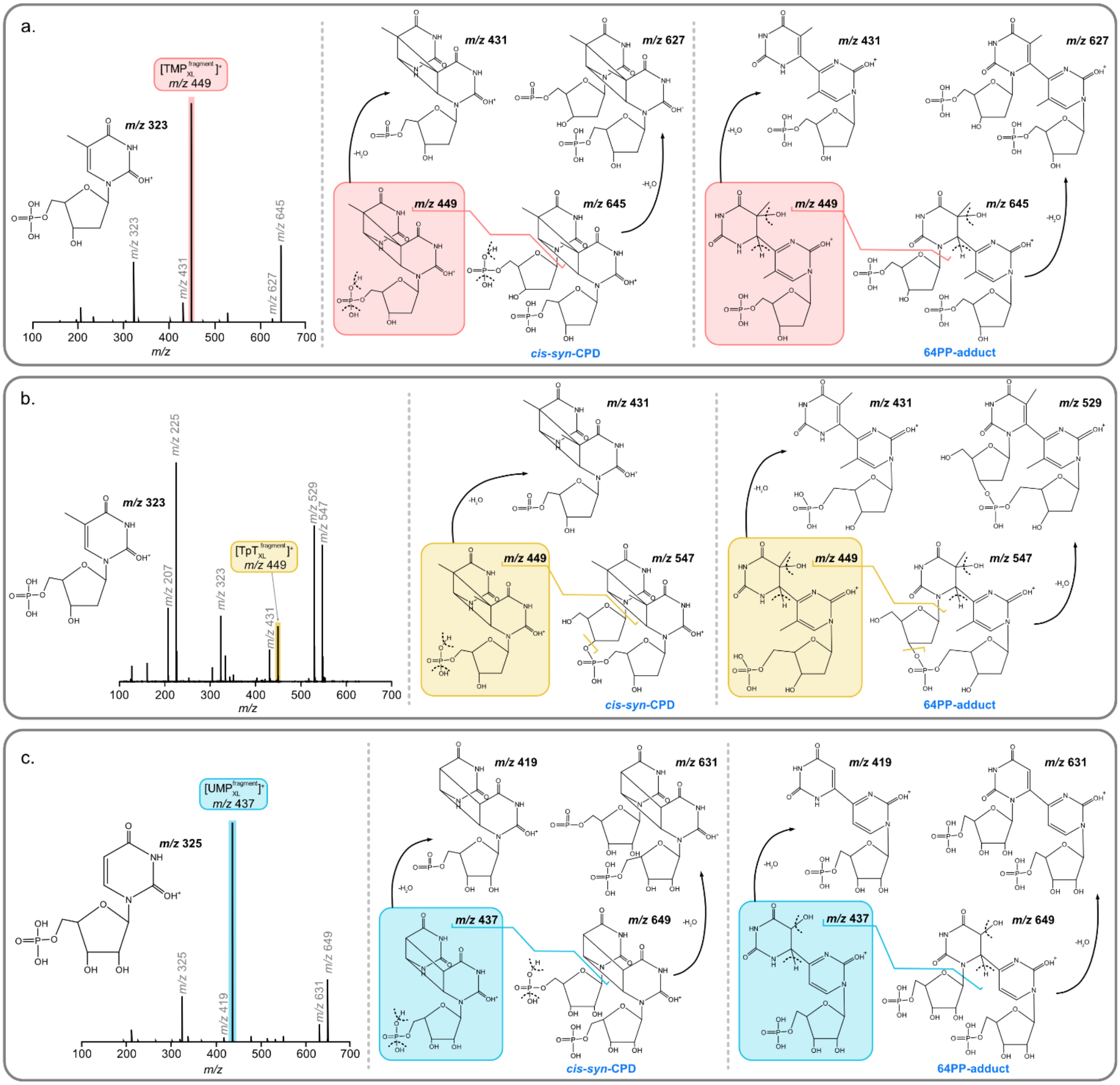

Figure S2. Tentative structural assignments of nucleotide fragments generated by tandem MS following UV irradiation at 254 nm: (a) TMP_{XL}; (b) TpT_{XL}; and (c) UMP_{XL}. Of the five isomers depicted in **Figure 1**, only the *cis*-*syn*-CPD and 64PP-adduct are illustrated here. Note that, for each isomer, the fragment ions at *m/z* 449 (for TMP_{XL}) and *m/z* 437 (for UMP_{XL}) can result from cleavage of a sugar-phosphate group linked to either of the thymine bases. Similarly, the fragment ion at *m/z* 449 for TpT_{XL} may arise from cleavage of a sugar group linked to either thymine base. To minimize computational costs, calculations were restricted to a single type of fragment ion.

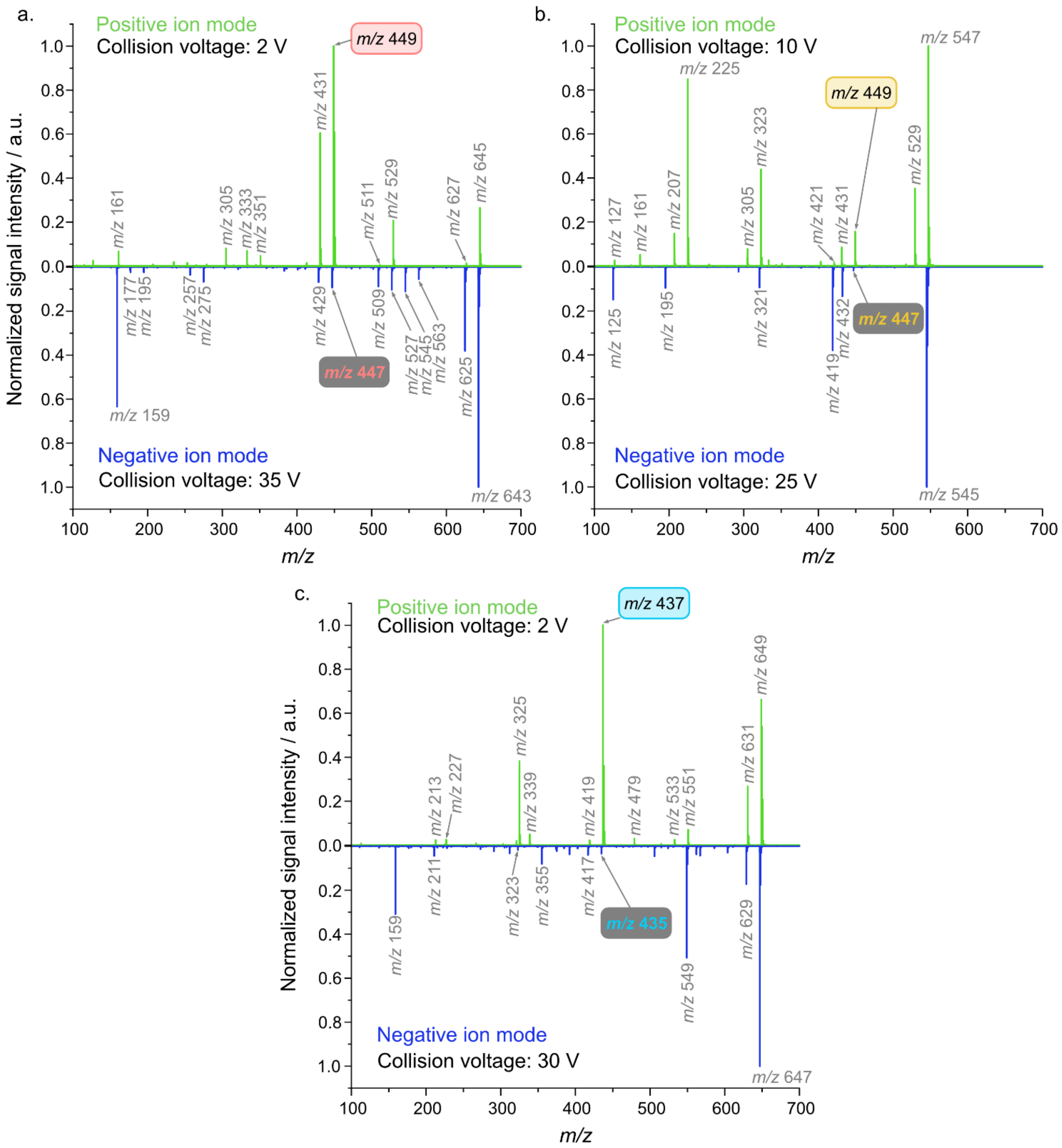

Figure S3. Comparison of tandem MS fragmentation patterns of nucleotide photoproducts in positive and negative ion modes. (a) TMP_{XL}; (b) TpT_{XL}; and (c) UMP_{XL}. Experiments were performed on a Synapt G2-S mass spectrometer (Waters, Manchester, U.K.). Voltages applied to the collision cell (collision gas: argon) and the ionization mode are specified in the respective plots.

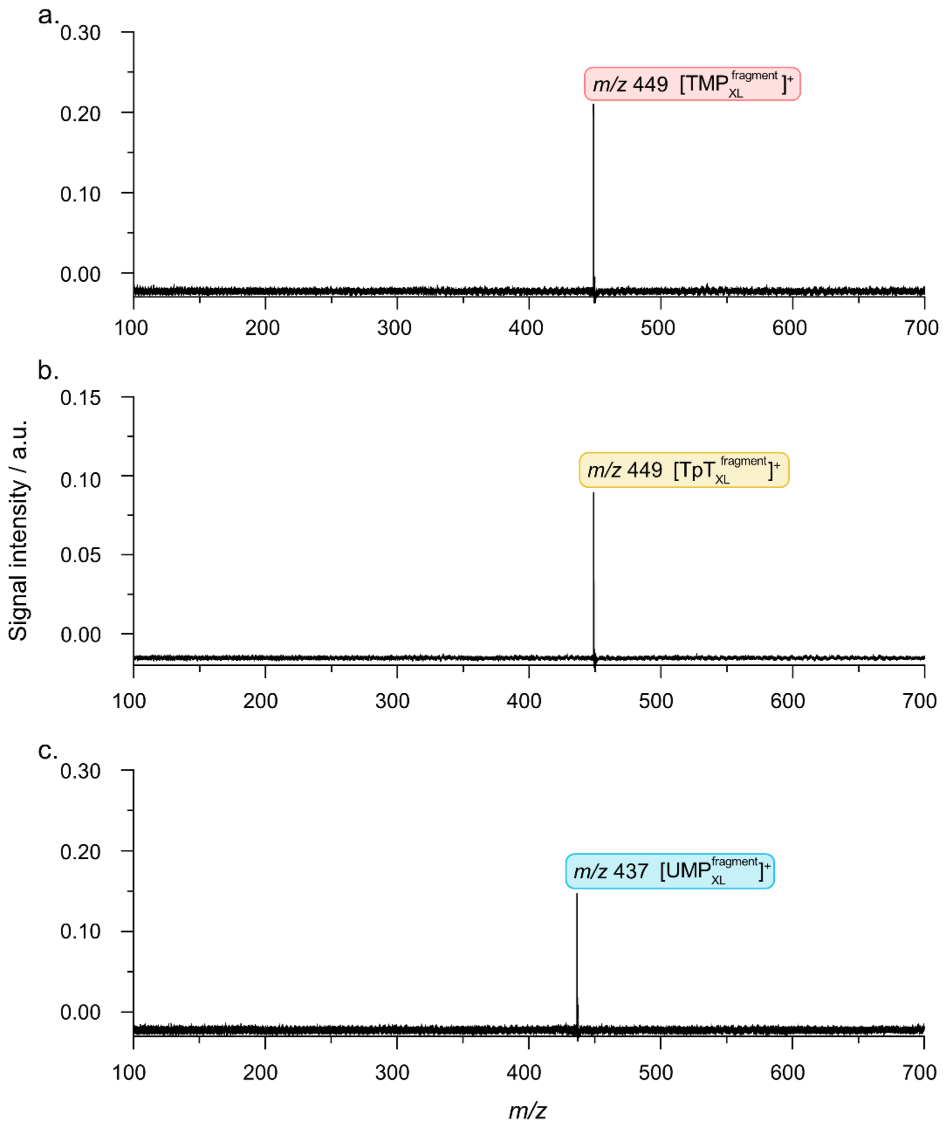

Figure S4. Mass spectra of fragment ions of photoproducts isolated using a quadrupole mass analyzer for cryogenic gas-phase IR ion spectroscopy: (a) TMP_{XL}; (b) TpT_{XL}; and (c) UMP_{XL}. Fragment ions were generated by in-source collision-induced dissociation.

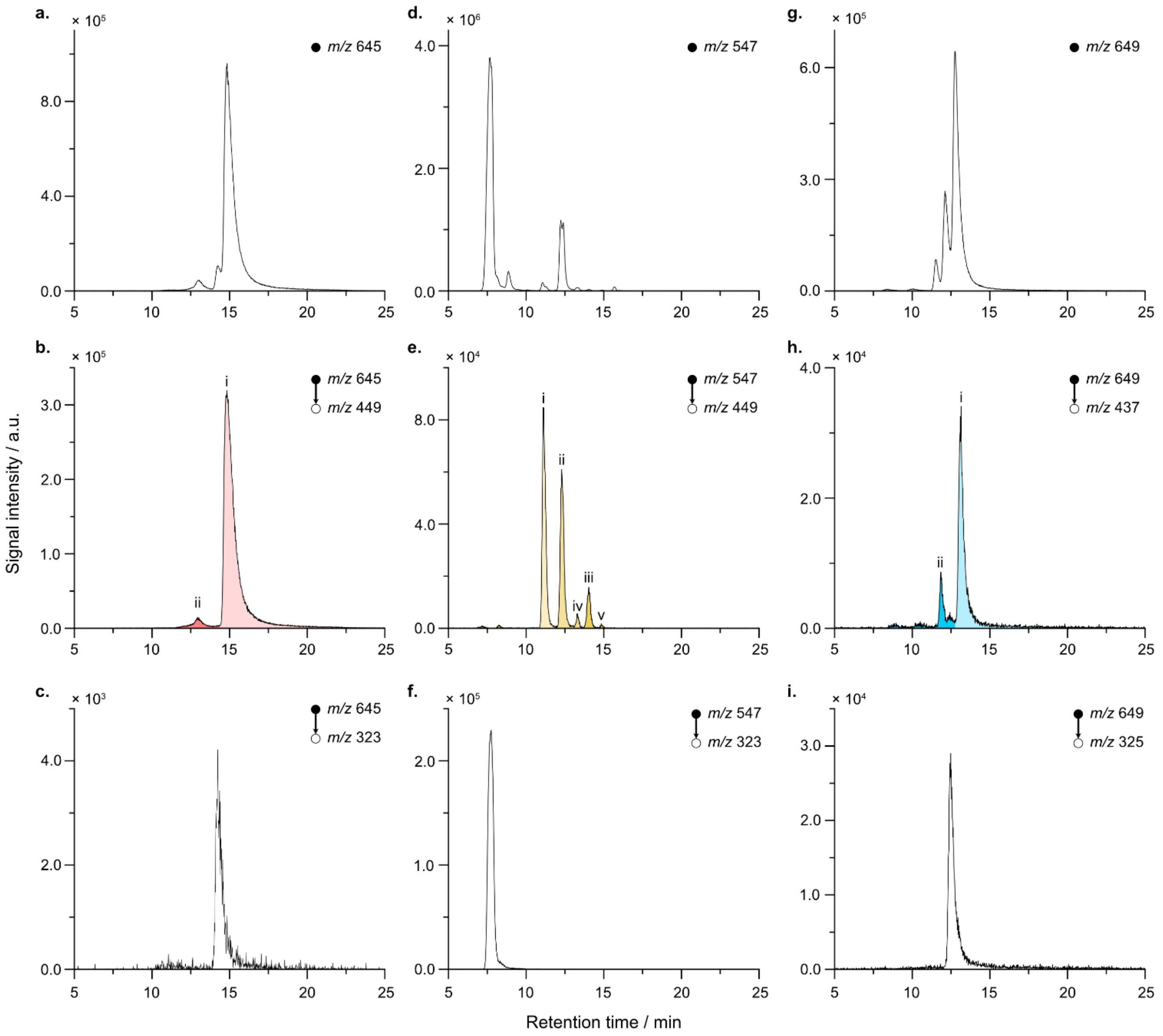

Figure S5. LC-MS/MS chromatograms of the photoproduct mixtures: panels (a–c) correspond to TMP_{XL}, (d–f) to TpT_{XL}, and (g–i) to UMP_{XL}. Top panel shows precursor ion chromatograms; middle panel displays fragment ion chromatograms corresponding to intact crosslinks; and bottom panel presents chromatograms of nucleotide monomers. Distinct color shades are employed to visually distinguish isobaric and isomeric photoproducts.

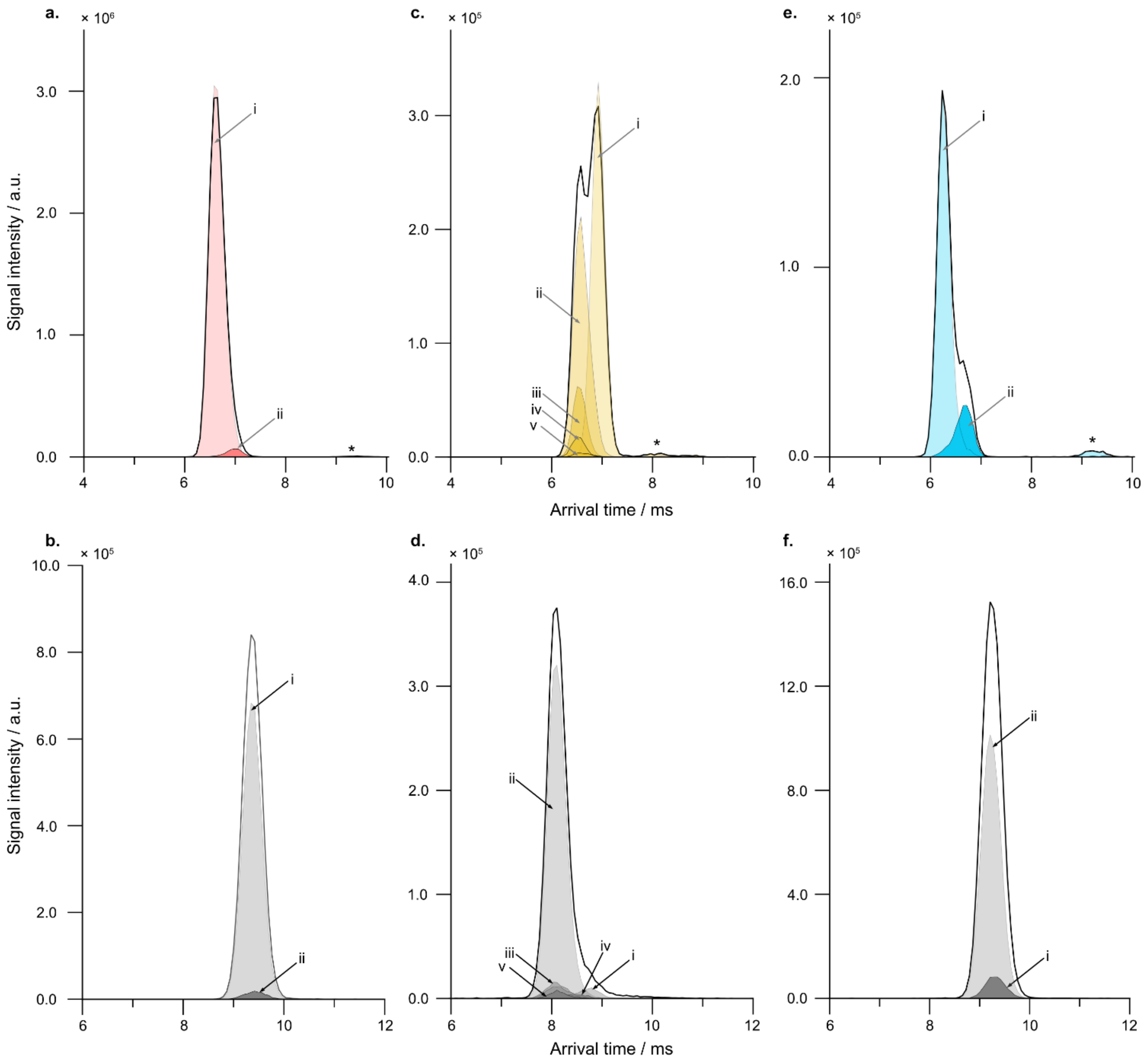

Figure S6. LC-MS/MS-TWIMS mobilograms of photoproduct fragment ions and their corresponding precursor ions: (a) TMP_{XL} fragment ion (*m/z* 449); (b) TMP_{XL} precursor ion (*m/z* 645); (c) TpT_{XL} fragment ion (*m/z* 449); (d) TpT_{XL} precursor ion (*m/z* 547); (e) UMP_{XL} fragment ion (*m/z* 437); and (f) UMP_{XL} precursor ion (*m/z* 649). Extracted-ion mobilograms of isobaric and isomeric photoproducts—distinguished by varying color shades—are overlaid to illustrate their individual contributions to the total-ion mobilogram, shown in bold black (see Figure S5). The extracted-ion-mobilograms of different isomers were obtained by processing the LC-MS/MS-TWIMS data using Driftscope software (Waters, Manchester, UK). The minor features highlighted with * could be attributed to aliasing (or phasing) caused due to mismatch between frequencies of Transfer T-Wave and ToF pusher in the Synapt G2-S mass spectrometer (Waters, Manchester, UK).

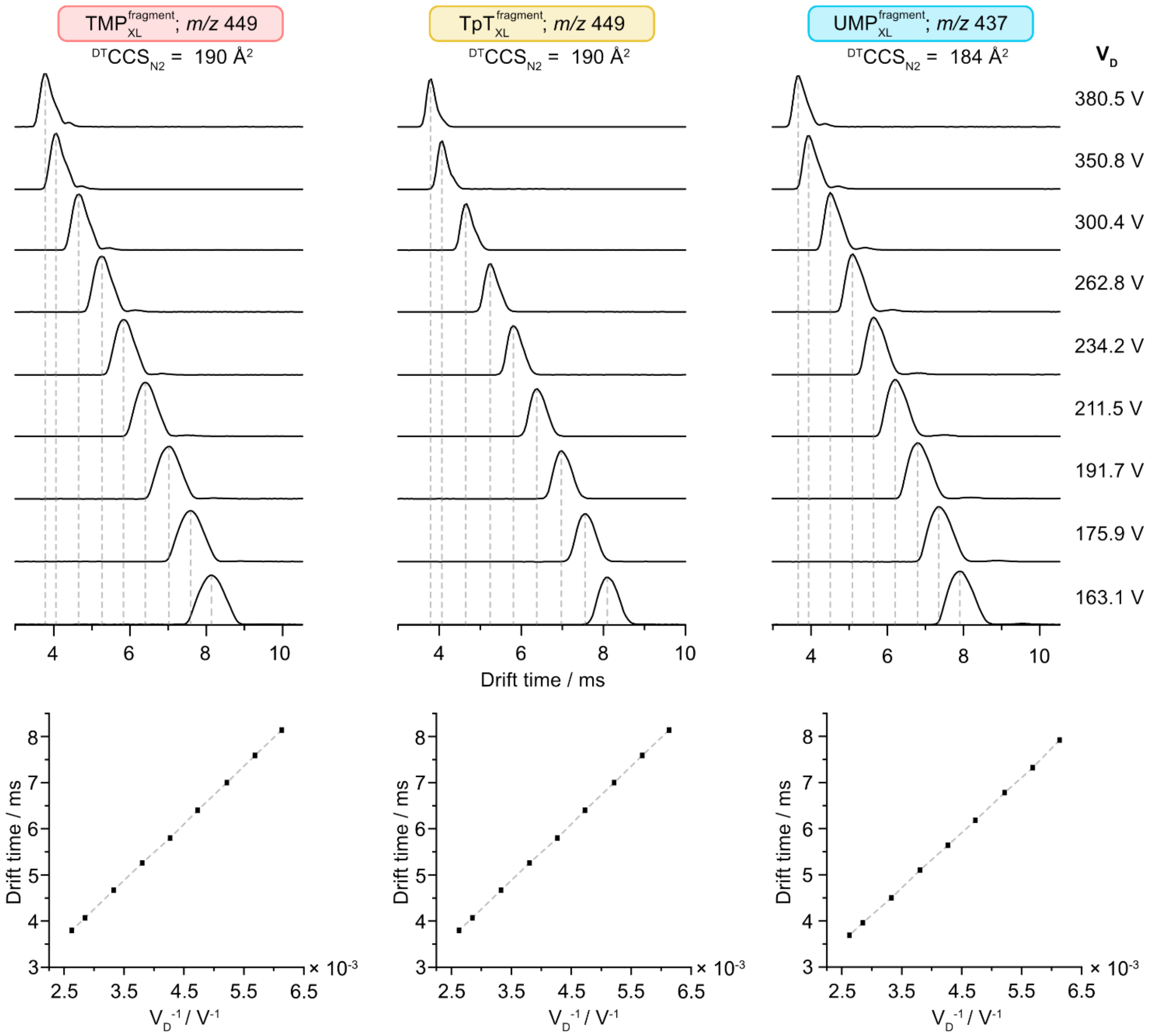

Figure S7. Drift-tube ion mobility-mass spectrometry of fragment ions of photoproducts. Top panel: Arrival time distributions recorded at different drift tube voltages (V_D) in the presence of nitrogen buffer gas. The specified $^{DT}CCS_{N2}$ values were calculated using the Mason–Schamp equation, following the determination of reduced ion mobilities from the slope of the linear regression of drift times (corresponding to peak maxima) plotted against the inverse of V_D (bottom panel). Owing to limited isomeric separation, the $^{DT}CCS_{N2}$ values obtained in this experiment allow only an approximate comparison between the size and shape of nucleotide photoproduct fragment ions and their predicted counterparts. This limitation is underscored by the LC-IMS-MS/MS data, which reveal the presence of multiple photoproducts capable of producing isobaric fragment ions (see **Figures S5** and **S6**).

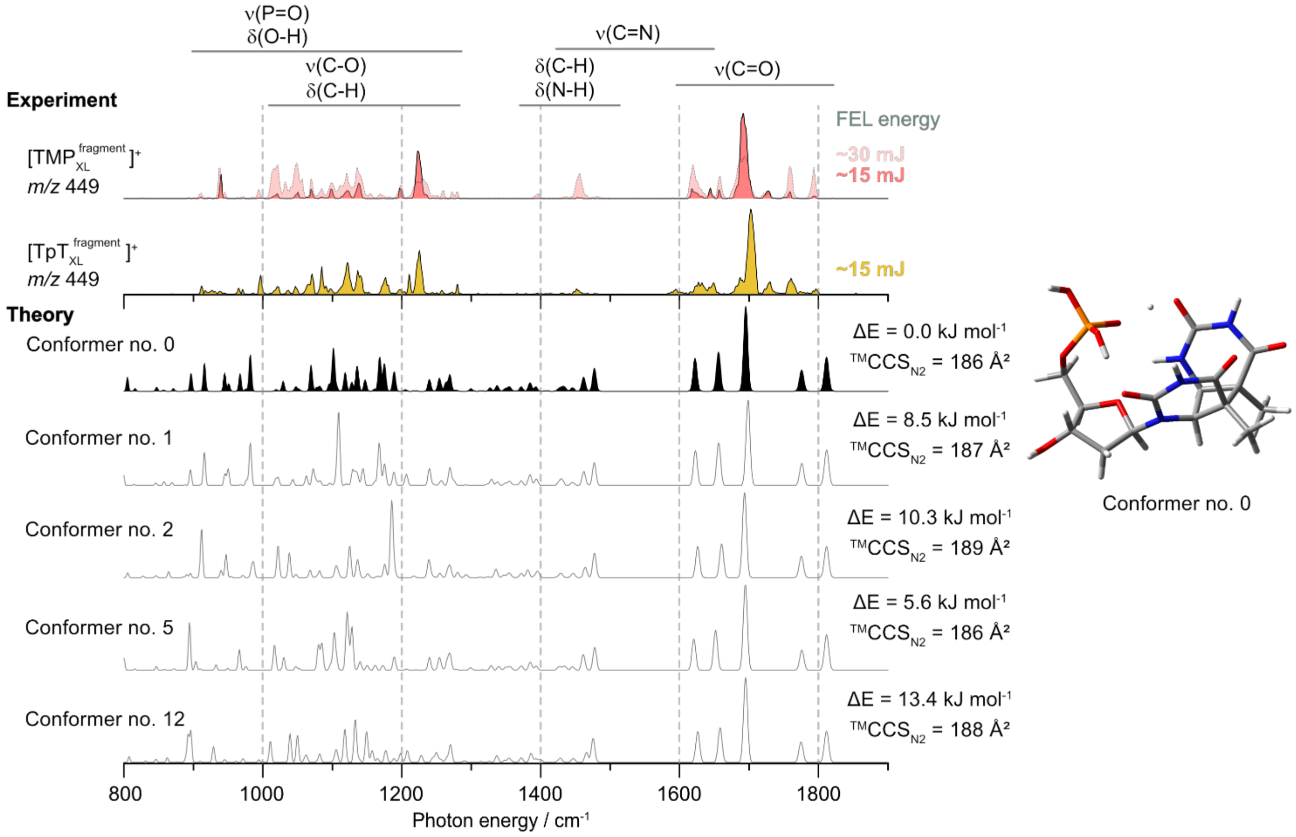

Figure S8. Comparison of experimental IR spectra of TMP_{XL} and TpT_{XL} fragment ions at *m/z* 449 with DFT calculated spectra of conformers of *cis*-*syn*-CPD isomer. Predicted transitions for the lowest-energy conformer no. 0 are highlighted, as they closely match the experimental data. Geometry optimized structure of conformer no. 0 at PBE0/6-311+G(d,p) with GD3BJ level of theory is shown. ΔE values (in kJ mol^{-1}) correspond to the sum of electronic and zero-point vibrational energies. See **Table S1** for more information.

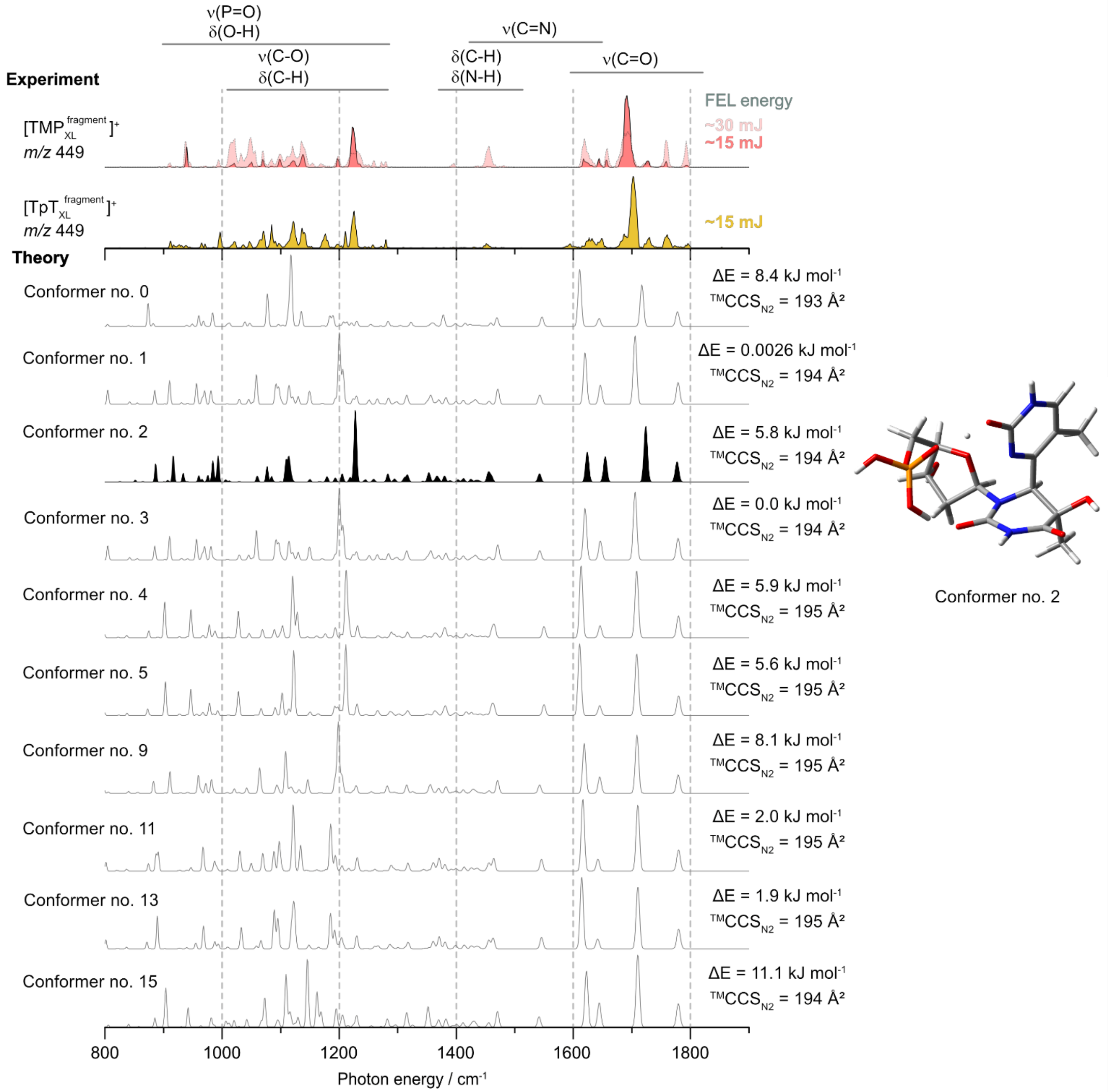

Figure S9. Comparison of experimental IR spectra of TMP_{XL} and TpT_{XL} fragment ions at *m/z* 449 with DFT calculated spectra of conformers of 64PP-adduct isomer. Predicted transitions for the conformer no. 2 are highlighted, as they closely match the experimental data. Geometry optimized structure of conformer no. 2 at PBE0/6-311+G(d,p) with GD3BJ level of theory is shown. ΔE values (in kJ mol^{-1}) correspond to the sum of electronic and zero-point vibrational energies. See **Table S1** for more information.

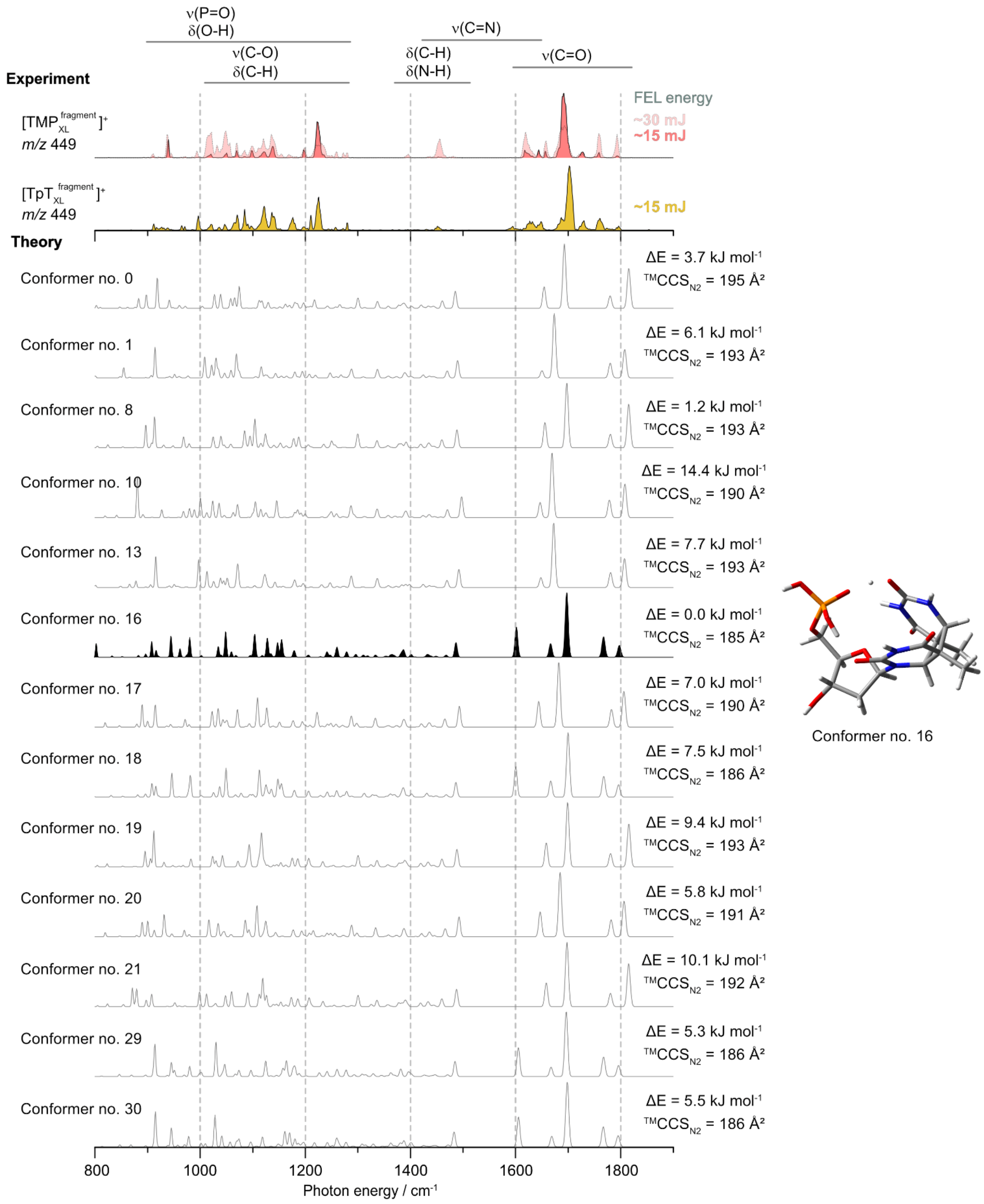

Figure S10. Comparison of experimental IR spectra of TMP_{XL} and TpT_{XL} fragment ions at *m/z* 449 with DFT calculated spectra of conformers of *cis*-*anti*-CPD isomer. Predicted transitions for the lowest-energy conformer no. 16 are highlighted, as they closely match the experimental data. Geometry optimized structure of conformer no. 16 at PBE0/6-311+G(d,p) with GD3BJ level of theory is shown. ΔE values (in kJ mol^{-1}) correspond to the sum of electronic and zero-point vibrational energies. See **Table S1** for more information.

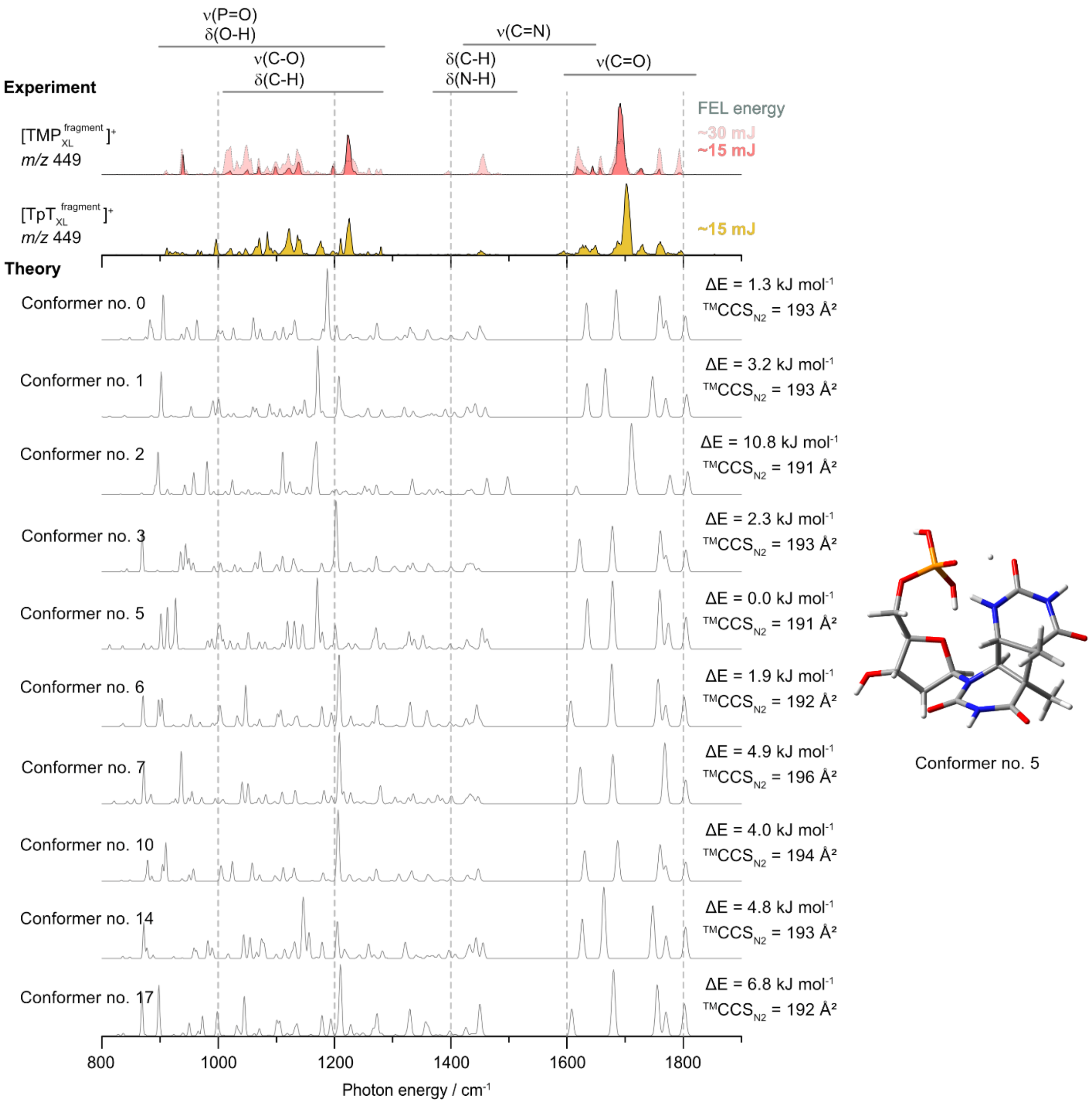

Figure S11. Comparison of experimental IR spectra of TMP_{XL} and TpT_{XL} fragment ions at *m/z* 449 with DFT calculated spectra of conformers of *trans-syn*-CPD isomer. The prominent transition around 1675 cm^{-1} predicted for most conformers do not match with the experiment. Geometry optimized structure of the lowest energy conformer no. 5 at PBE0/6-311+G(d,p) with GD3BJ level of theory is shown. ΔE values (in kJ mol^{-1}) correspond to the sum of electronic and zero-point vibrational energies. See **Table S1** for more information.

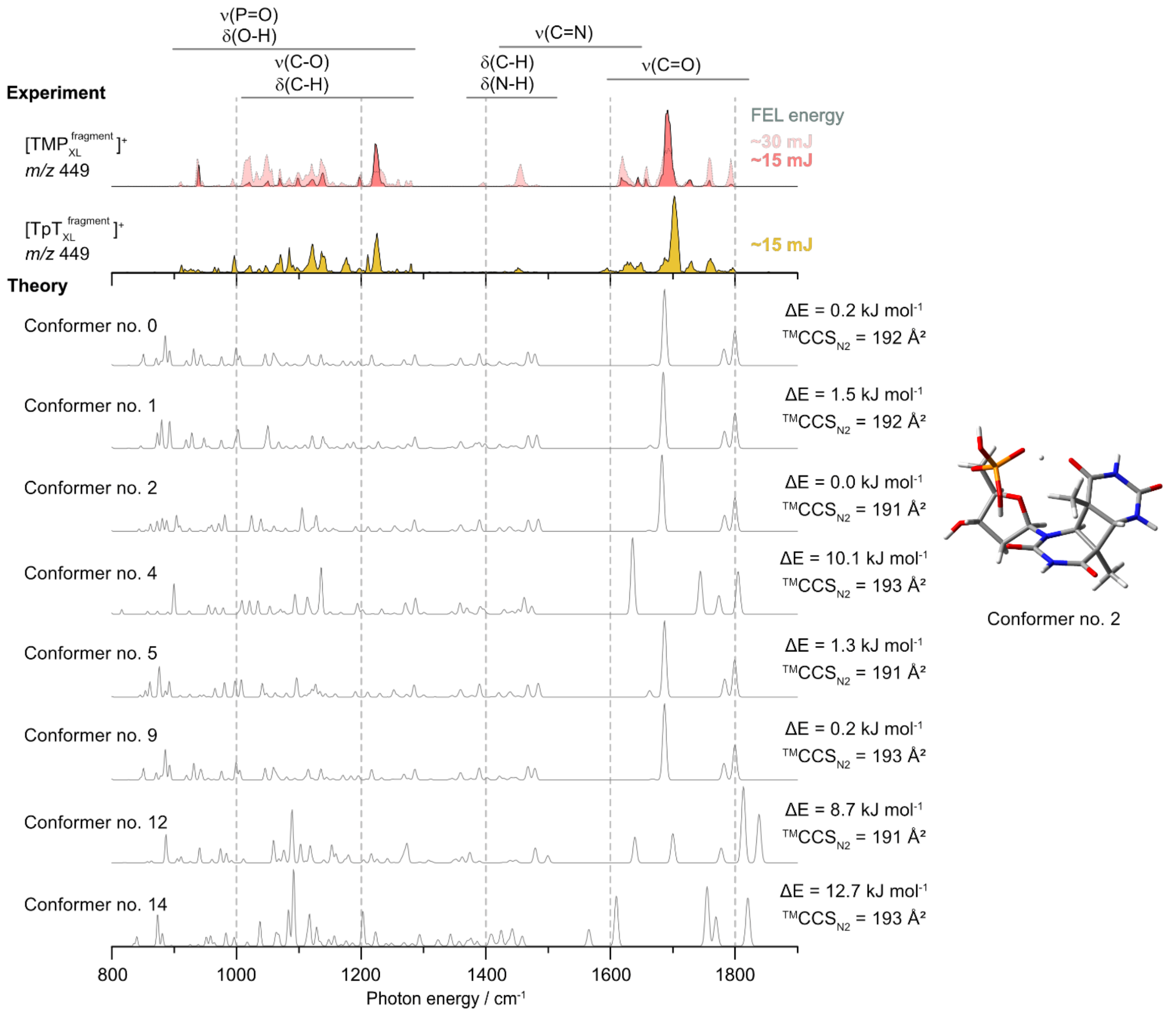

Figure S12. Comparison of experimental IR spectra of TMP_{XL} and TpT_{XL} fragment ions at *m/z* 449 with DFT calculated spectra of conformers of *trans-anti*-CPD isomer. The prominent transition around 1680 cm^{-1} predicted for most conformers do not match with the experiment. Geometry optimized structure of the lowest energy conformer no. 2 at PBE0/6-311+G(d,p) with GD3BJ level of theory is shown. ΔE values (in kJ mol^{-1}) correspond to the sum of electronic and zero-point vibrational energies. See **Table S1** for more information.

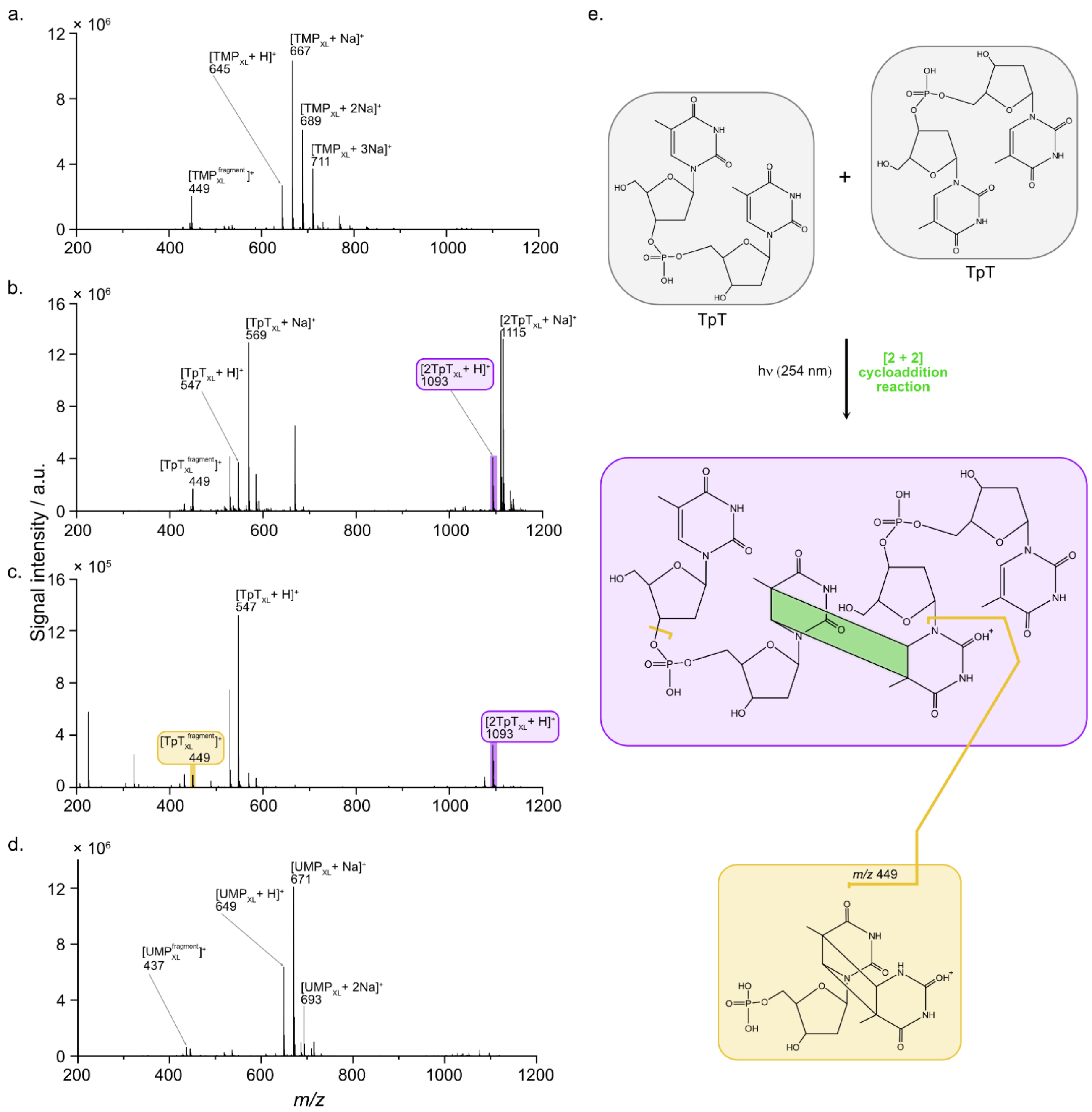

Figure S13. Mass spectra of nucleotides after UV (254 nm) irradiation. (a) Mass spectrum of TMP_{XL}; (b) mass spectrum of TpT_{XL}; (c) tandem MS spectrum of precursor ion *m/z* 1093 $[2TpT_{XL}+H]^+$; (d) mass spectrum of UMP_{XL}; (e) putative mechanism of formation of *cis-anti*-CPD isomer upon UV irradiation of TpT solution.

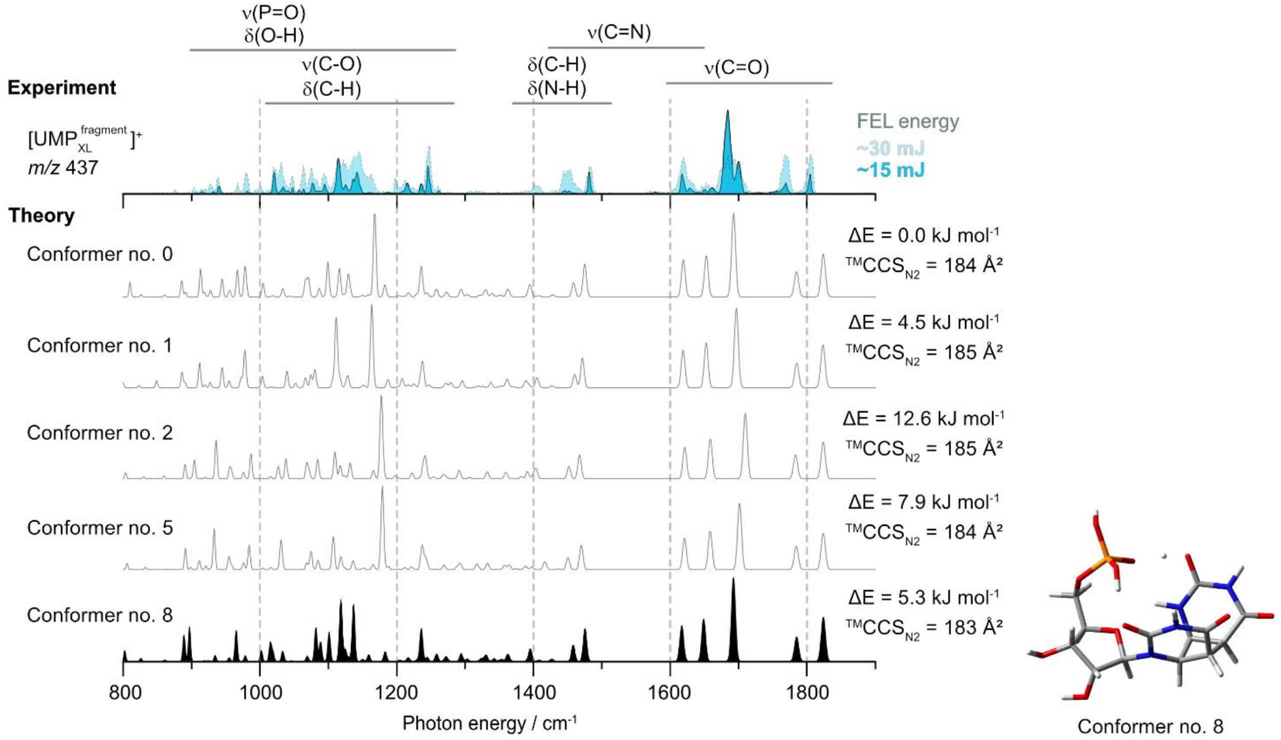

Figure S14. Comparison of experimental IR spectrum of UMP_{XL} fragment ion at *m/z* 437 with DFT calculated spectra of conformers of *cis*-*syn*-CPD isomer. Predicted transitions for the conformer no. 8 are highlighted, as they closely match the experimental data. Geometry optimized structure of conformer no. 8 at PBE0/6-311+G(d,p) with GD3BJ level of theory is shown. ΔE values (in kJ mol^{-1}) correspond to the sum of electronic and zero-point vibrational energies. See **Table S1** for more information.

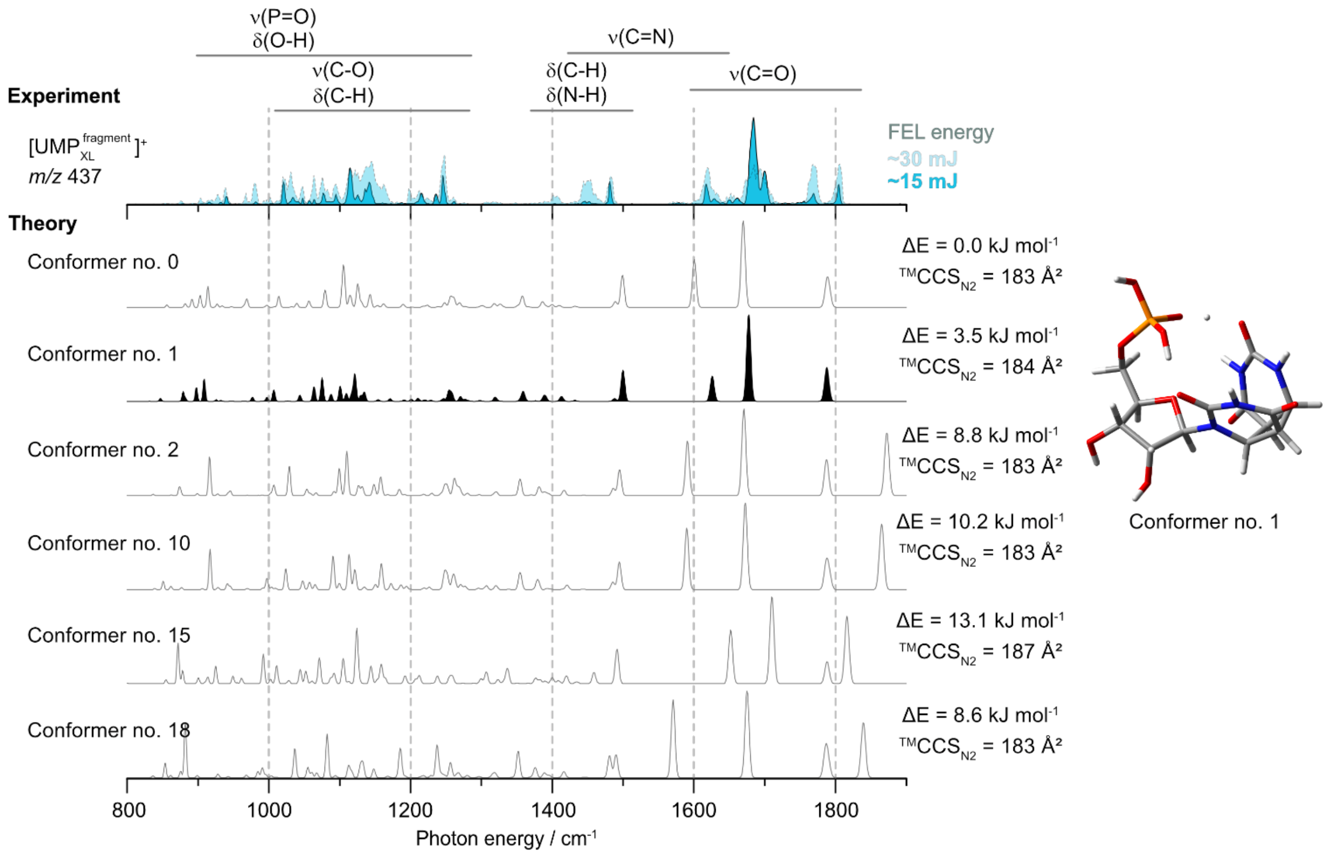

Figure S15. Comparison of experimental IR spectrum of UMP$_{XL}$ fragment ion at *m/z* 437 with DFT calculated spectra of conformers of *cis*-*anti*-CPD isomer. Predicted transitions for the conformer no. 1 are highlighted, as they closely match the experimental data. Geometry optimized structure of conformer no. 1 at PBE0/6-311+G(d,p) with GD3BJ level of theory is shown. ΔE values (in kJ mol^{-1}) correspond to the sum of electronic and zero-point vibrational energies. See **Table S1** for more information.

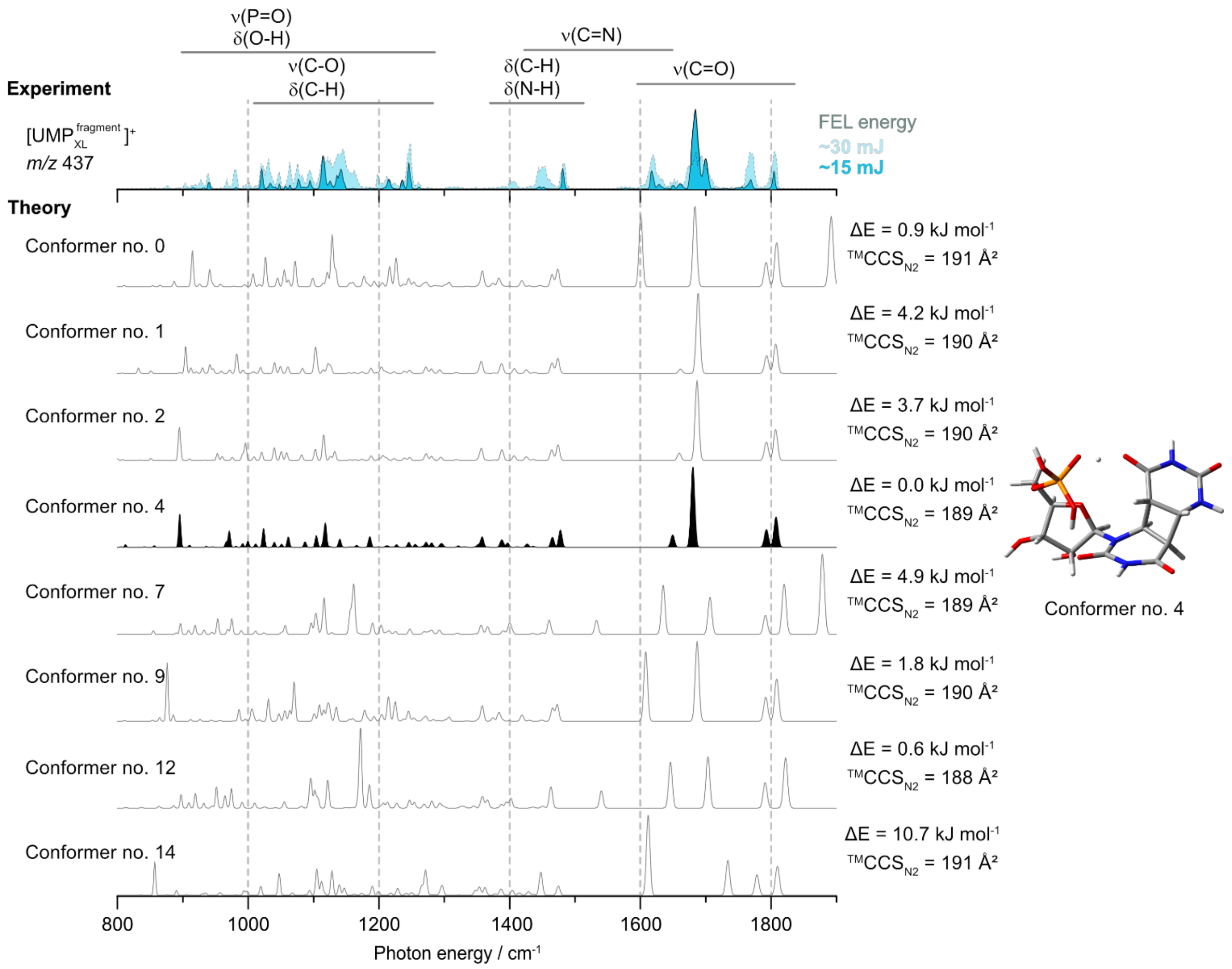

Figure S16. Comparison of experimental IR spectrum of UMP$_{XL}$ fragment ion at *m/z* 437 with DFT calculated spectra of conformers of *trans*-*anti*-CPD isomer. Predicted transitions for the lowest-energy conformer no. 4 are highlighted, as they closely match the experimental data. Geometry optimized structure of conformer no. 4 at PBE0/6-311+G(d,p) with GD3BJ level of theory is shown. ΔE values (in kJ mol^{-1}) correspond to the sum of electronic and zero-point vibrational energies. See **Table S1** for more information.

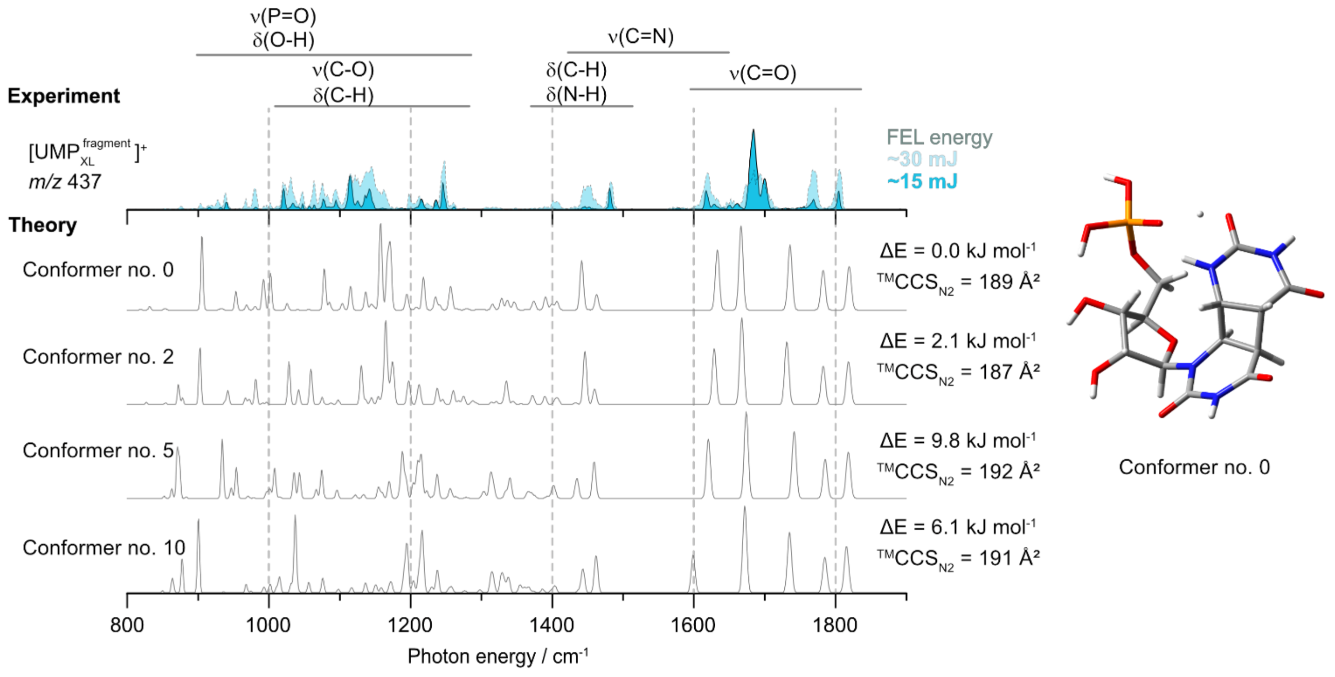

Figure S17. Comparison of experimental IR spectrum of UMP$_{XL}$ fragment ion at *m/z* 437 with DFT calculated spectra of conformers of *trans-syn*-CPD isomer. The prominent transition around 1730 cm^{-1} predicted for all the conformers do not match with the experimental data. Geometry optimized structure of conformer no. 0 at PBE0/6-311+G(d,p) with GD3BJ level of theory is shown. ΔE values (in kJ mol^{-1}) correspond to the sum of electronic and zero-point vibrational energies. See **Table S1** for more information.

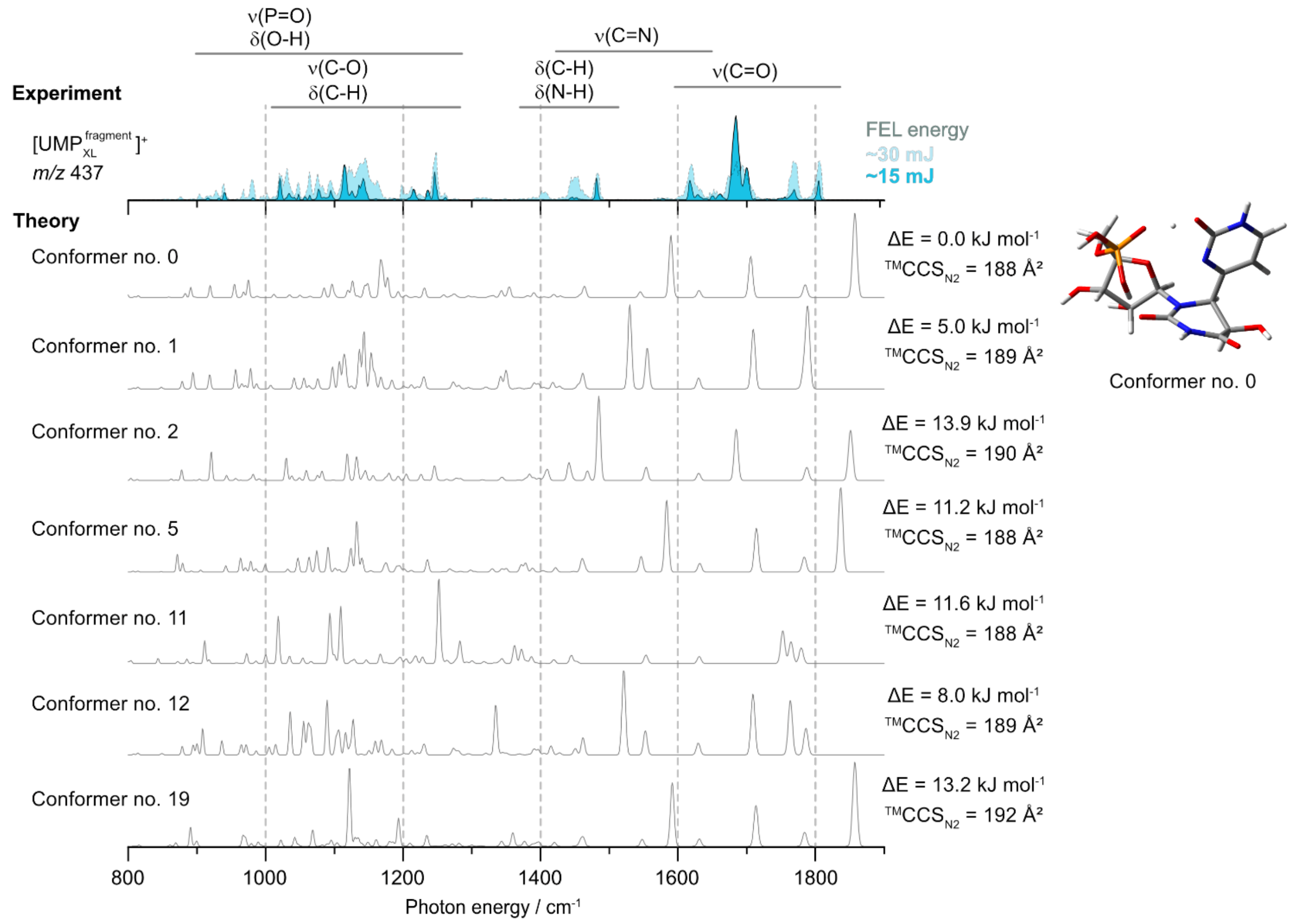

Figure S18. Comparison of experimental IR spectrum of UMP_{XL} fragment ion at *m/z* 437 with DFT calculated spectra of conformers of 64PP-adduct isomer. The prominent transitions predicted for all the conformers do not match with the experimental data. Geometry optimized structure of conformer no. 0 at PBE0/6-311+G(d,p) with GD3BJ level of theory is shown. ΔE values (in kJ mol^{-1}) correspond to the sum of electronic and zero-point vibrational energies. See **Table S1** for more information.

Table S2. Comparison of experimental $^{DT}CCS_{N2}$ values with theoretical $^{TM}CCS_{N2}$ values calculated using HPCCS algorithm.[3] Note that the experimental CCS values were obtained for unresolved mobilogram peaks containing two or more isomers (see **Figures S6** and **S7**), and the $^{DT}CCS_{N2}$ values obtained in this experiment allow only an approximate comparison between the size and shape of nucleotide photoproduct fragment ions and their predicted counterparts. Values shown here correspond to the conformers for which experimental IR spectra matches with theory. Relative electronic energies (ΔE) specified are sum of electronic and zero-point vibrational energies. The theoretical $^{TM}CCS_{N2}$ values of other conformers are shown alongside the theoretical spectra in **Figures S8-S12** and **S14-S18** as well as in **Table S1**.

<table>
<tr><th>Photoproduct fragment ion</th><th>Experiment $^{DT}CCS_{N2}$ (Å^2)</th><th colspan="4">Theory $^{TM}CCS_{N2}$ (Å^2)</th></tr>
<tr><td colspan="2">Isomer</td><td>cis-syn-CPD</td><td>cis-anti-CPD</td><td>trans-anti-CPD</td><td>64PP-adduct</td></tr>
<tr><td colspan="2">Conformer no.</td><td>0</td><td>16</td><td>-</td><td>2</td></tr>
<tr><td colspan="2">ΔE (kJ mol^{-1})</td><td>0.0</td><td>0.0</td><td>-</td><td>5.8</td></tr>
<tr><td>TMP_{XL} (m/z 449)</td><td>190</td><td rowspan="2">186</td><td rowspan="2">185</td><td rowspan="2">-</td><td rowspan="2">194</td></tr>
<tr><td>TpT_{XL} (m/z 449)</td><td>190</td></tr>
<tr><td colspan="2">Conformer no.</td><td>8</td><td>1</td><td>4</td><td>-</td></tr>
<tr><td colspan="2">ΔE (kJ mol^{-1})</td><td>5.3</td><td>3.5</td><td>0.0</td><td>-</td></tr>
<tr><td>UMP_{XL} (m/z 437)</td><td>184</td><td>183</td><td>184</td><td>189</td><td>-</td></tr>
</table>

Atomic XYZ coordinates of optimized structures of the conformers whose theoretical spectra match the experimental data.

Conformer no. 0 of fragment ion at *m/z* 449 of *cis*-*syn*-CPD isomer of TMP_{XL} and TpT_{XL}.

```
52
Charge =  1 Multiplicity = 1
C         -2.84640       -1.23020       -1.25870
C         -3.56780        0.08470       -1.39810
H         -3.10440        0.70390       -2.16890
H         -4.60930       -0.10620       -1.65810
O         -3.59400        0.79070       -0.14510
P         -2.60940        1.95630        0.26220
O         -1.45920        2.06270       -0.69280
O         -2.26910        1.68990        1.76860
H         -1.91340        0.78040        1.94960
O         -3.39660        3.32100        0.27610
H         -4.07220        3.41610        0.95680
C         -3.26540       -2.08400       -0.06280
O         -4.38500       -2.83660       -0.45350
H         -4.87720       -3.11370        0.32160
C         -2.00760       -2.90910        0.22880
C         -0.87260       -2.13650       -0.44880
N          0.22010       -1.65690        0.38270
C         -0.06010       -0.85740        1.44420
O         -1.19680       -0.69120        1.87520
N          1.00220       -0.21280        2.04750
C          2.30660       -0.09730        1.59390
O          3.05770        0.71660        2.06310
C          2.71750       -1.10520        0.54150
C          3.61710       -2.12310        1.22120
H          3.92710       -2.89980        0.52040
H          4.50530       -1.63080        1.62240
H          3.08980       -2.60460        2.04990
C          1.50860       -1.61170       -0.26090
H          1.72230       -2.59590       -0.68130
C          1.77210       -0.51630       -1.33280
N          0.99970        0.67820       -1.06690
C          1.53030        1.86390       -0.81650
N          2.86530        2.01140       -0.72000
C          3.80340        0.96800       -0.66950
O          4.96810        1.20910       -0.56030
C          3.21930       -0.42200       -0.79410
C          4.18320       -1.28580       -1.59180
H          3.80120       -2.30000       -1.72690
H          4.34100       -0.84920       -2.58090
H          5.14960       -1.33540       -1.08950
H          3.21000        2.94440       -0.52450
O          0.82520        2.91470       -0.64690
H         -0.19330        2.68750       -0.66460
H         -0.02070        0.62100       -1.13270
H          1.65860       -0.80990       -2.37860
H          0.74970        0.38540        2.82540
H         -0.38890       -2.76660       -1.20280
O         -1.44950       -1.00920       -1.09760
H         -1.83140       -3.05130        1.29330
H         -2.11560       -3.88740       -0.24470
H         -3.49600       -1.43380        0.78470
H         -3.03640       -1.80870       -2.17470
```

Conformer no. 2 of fragment ion at *m/z* 449 of 64PP-adduct isomer of TMP_{XL} and TpT_{XL}.

```
52
Charge =  1 Multiplicity = 1
C          2.02360        1.36530       -1.71230
C          2.26310        2.74540       -1.08640
C          1.35000        2.73570        0.13420
C          0.18940        1.90100       -0.38520
N         -0.72020        1.26100        0.55360
C         -0.28010        0.66700        1.70240
O          0.86240        0.73060        2.12460
N         -1.22900       -0.00150        2.46150
C         -2.55480       -0.16060        2.16950
O         -3.28630       -0.91260        2.76450
C         -3.07370        0.72880        1.05070
C         -3.41280        2.10260        1.62330
H         -2.53050        2.61770        2.00890
H         -3.86640        2.70980        0.83640
H         -4.13720        1.99120        2.43290
C         -1.98340        0.83990       -0.03440
H         -2.29670        1.62770       -0.72430
C         -1.84150       -0.45390       -0.82020
N         -0.92870       -1.29120       -0.36720
C         -0.71530       -2.43070       -1.00870
N         -1.51560       -2.78200       -2.04500
C         -2.46040       -1.94300       -2.52570
C         -2.64900       -0.71220       -1.95920
C         -3.63380        0.26030       -2.52450
H         -3.13190        1.17860       -2.84560
H         -4.39060        0.51870       -1.78250
H         -4.13350       -0.16100       -3.39810
H         -3.03030       -2.29960       -3.37540
H         -1.33750       -3.67580       -2.48940
O          0.22210       -3.24800       -0.71810
H          1.02460       -2.78090       -0.23690
O         -4.20180        0.11790        0.49300
H         -4.58870       -0.43430        1.18940
H         -0.85890       -0.50380        3.25900
H         -0.45430        2.54460       -0.99910
O          0.76670        0.90940       -1.21720
H          1.84630        2.24790        0.96760
H          1.03670        3.73900        0.42870
H          3.31300        2.88600       -0.80940
O          1.85890        3.69140       -2.05860
H          2.08980        4.57280       -1.75600
C          3.07150        0.31460       -1.43250
H          4.02310        0.59870       -1.88920
H          2.74410       -0.64250       -1.84660
O          3.27780        0.19150       -0.01900
P          3.18670       -1.20560        0.74360
O          2.20820       -2.13300        0.12670
O          2.97980       -0.78590        2.23760
H          2.18990       -0.21000        2.36820
O          4.61680       -1.88230        0.72720
H          5.28700       -1.43340        1.25370
H          1.96260        1.49620       -2.79910
```

Conformer no. 16 of fragment ion at *m/z* 449 of *cis-anti*-CPD isomer of TMP_{XL} and TpT_{XL}.

```
52
Charge =  1 Multiplicity = 1
C         -2.32750        1.79150        0.55100
C         -3.37120        0.79160        0.97280
H         -3.13300        0.37640        1.95460
H         -4.34590        1.27850        1.01170
O         -3.48810       -0.26530        0.00630
P         -2.82220       -1.69500        0.07740
O         -2.27280       -1.95270       -1.36850
H         -1.72660       -1.21180       -1.73990
O         -3.96030       -2.76820        0.26520
H         -4.60270       -2.83490       -0.44990
O         -1.83630       -1.84310        1.20100
H         -0.59350       -2.53060        1.14100
C         -2.45560        2.36510       -0.85770
C         -1.00770        2.76080       -1.18220
H         -0.74060        2.57190       -2.22080
C         -0.15850        1.93810       -0.20030
N          0.84060        1.03010       -0.76210
C          0.42980        0.11640       -1.67110
O         -0.73040        0.03990       -2.06310
N          1.36330       -0.77880       -2.16250
C          2.65300       -0.97260       -1.73290
O          3.30240       -1.92670       -2.08150
C          3.18280        0.04620       -0.75560
C          4.52640        0.55250       -1.25830
H          4.39710        1.11790       -2.18490
H          5.18360       -0.29490       -1.46050
H          5.00880        1.20390       -0.52850
C          2.20940        1.15800       -0.28910
H          2.56370        2.17010       -0.50350
C          2.51070        0.75150        1.21210
C          1.28220        0.55070        2.06420
O          0.83770        1.37540        2.80950
N          0.62190       -0.66520        1.87410
C          1.13700       -1.73080        1.23900
O          0.42350       -2.76990        1.03190
N          2.40080       -1.72930        0.84200
H          2.70580       -2.56750        0.35960
C          3.21010       -0.53200        0.69880
H          4.20570       -0.71750        1.10420
H         -0.37050       -0.69520        2.10280
C          3.45010        1.72470        1.90150
H          4.31330        1.95980        1.27830
H          3.81140        1.30390        2.84280
H          2.92020        2.65100        2.12910
H          0.99540       -1.45760       -2.81920
H          0.41060        2.60440        0.45820
O         -1.05470        1.17190        0.57910
H         -0.88220        3.82570       -0.97410
H         -2.79630        1.58190       -1.54060
O         -3.36160        3.43620       -0.80460
H         -3.70970        3.61210       -1.68080
H         -2.36910        2.62800        1.26580
```

Conformer no. 8 of fragment ion at *m/z* 437 of *cis*-*syn*-CPD isomer of UMP_{XL}.

```
47
Charge =  1 Multiplicity = 1
C         -2.71490       -0.60480       -1.17810
C         -3.27300       -1.30400        0.05990
O         -4.56650       -1.78820       -0.20740
H         -5.13700       -1.63650        0.54890
H         -3.25820       -0.62300        0.91130
C         -2.26900       -2.44980        0.28300
H         -2.11370       -2.66460        1.34420
O         -2.65980       -3.59560       -0.42340
H         -3.62630       -3.60600       -0.44020
C         -0.98930       -1.93820       -0.39480
N          0.19530       -1.73610        0.41420
C          0.12370       -0.89850        1.48090
O         -0.94370       -0.47930        1.91800
N          1.30840       -0.51220        2.07510
C          2.59970       -0.69380        1.60520
O          3.52700       -0.07750        2.05590
C          2.73950       -1.73450        0.52470
H          3.24260       -2.59480        0.97010
C          1.44390       -2.00730       -0.24870
H          1.42870       -3.02600       -0.64060
C          1.92460       -1.02430       -1.35850
N          1.44930        0.32170       -1.10850
C          2.24150        1.36580       -0.91110
O          1.79420        2.55150       -0.76920
N          3.57880        1.21310       -0.84440
C          4.26050       -0.01510       -0.79060
O          5.45150       -0.05500       -0.74520
C          3.35190       -1.21260       -0.80890
H          3.87690       -2.01730       -1.32250
H          4.13040        2.05290       -0.70850
H          0.44160        0.49220       -1.16640
H          1.73310       -1.30630       -2.39500
H          1.20490        0.11860        2.86170
H         -0.71850       -2.68780       -1.14480
O         -1.30020       -0.70540       -1.03770
C         -3.11040        0.83790       -1.34480
H         -4.17070        0.89400       -1.59420
H         -2.52250        1.30720       -2.13670
O         -2.95460        1.56260       -0.11460
P         -1.71420        2.46520        0.26940
O         -1.33810        2.09510        1.73460
H         -1.25190        1.12320        1.91650
O         -2.21590        3.94620        0.46870
H         -2.23290        4.48770       -0.32700
O         -0.63210        2.34030       -0.77260
H          0.74510        2.57100       -0.76640
H         -3.04850       -1.15160       -2.07110
```

Conformer no. 1 of fragment ion at *m/z* 437 of *cis*-*anti*-CPD isomer of UMP_{XL}.

47
Charge = 1 Multiplicity = 1

C	-2.18560	1.27400	0.77550
C	-3.21480	0.20310	1.00200
H	-2.99940	-0.37300	1.90360
H	-4.20380	0.65430	1.08000
O	-3.28520	-0.69280	-0.12830
P	-2.42340	-1.98130	-0.35340
O	-3.39710	-3.17630	-0.63180
O	-1.65670	-2.33010	0.92400
H	-0.63490	-2.62380	0.89200
O	-1.57080	-1.82250	-1.63510
H	-1.04930	-0.97730	-1.80900
C	-2.34800	2.11700	-0.49450
O	-3.29980	3.12430	-0.38660
H	-2.92100	3.83180	0.14880
H	-2.65140	1.46720	-1.31780
C	-0.90780	2.62480	-0.74420
O	-0.73170	3.93180	-0.23980
H	-0.87620	4.56800	-0.94430
H	-0.63680	2.56590	-1.79820
C	-0.05300	1.69220	0.13660
N	1.11880	1.08170	-0.45960
C	0.96160	0.39150	-1.61280
O	-0.14520	0.21550	-2.12610
N	2.07810	-0.12160	-2.23130
C	3.36570	-0.18760	-1.72990
O	4.23440	-0.80030	-2.28950
C	3.54800	0.48920	-0.40650
H	4.49920	1.02170	-0.41400
C	2.40910	1.36120	0.15490
H	2.58270	2.43850	0.15110
C	2.66070	0.67620	1.53980
C	1.47690	0.30210	2.38230
N	0.99000	-0.97180	2.14500
C	1.42000	-1.83590	1.17060
O	0.72940	-2.81150	0.83920
N	2.60440	-1.59340	0.59280
H	2.91110	-2.30390	-0.05890
C	3.44770	-0.44410	0.83640
H	4.39570	-0.74590	1.28280
H	0.10060	-1.19100	2.57600
O	0.95180	1.04270	3.16930
H	-3.89930	-3.15280	-1.45440
H	3.31660	1.30760	2.13920
H	1.89570	-0.61740	-3.09630
O	-0.90530	0.67730	0.63020
H	0.31130	2.30480	0.97000
H	-2.20150	1.93480	1.65720

Conformer no. 4 of fragment ion at *m/z* 437 of *trans*-*anti*-CPD isomer of UMP_{XL}.

```
47
Charge =  1 Multiplicity = 1
C        -2.58030       0.12230       1.46150
C        -3.31860      -1.13150       1.09250
H        -2.89870      -1.99820       1.60700
H        -4.37550      -1.03410       1.33880
O        -3.26990      -1.36220      -0.33360
P        -2.05080      -2.00630      -1.08670
O        -2.64240      -3.01490      -2.12860
H        -2.98660      -3.84630      -1.78300
O        -1.33380      -0.96780      -1.97930
H        -1.50560       0.01140      -1.85620
O        -1.13150      -2.77060      -0.13320
H        -0.26110      -2.42680       0.34090
H        -2.60720       0.21690       2.55680
C        -3.07830       1.42870       0.85320
O        -4.18320       1.90480       1.57870
H        -4.80950       2.32710       0.98680
C        -1.84020       2.32990       1.01620
O        -1.83420       2.92950       2.28330
H        -2.75070       3.13000       2.51400
C        -0.67520       1.32260       1.01090
N         0.31290       1.43990      -0.03270
C        -0.06950       1.59720      -1.32680
O        -1.24770       1.57660      -1.68670
N         0.92950       1.76190      -2.25580
C         2.31020       1.76410      -2.06490
O         3.06330       1.95770      -2.97760
C         2.76910       1.46190      -0.66220
H         3.38430       2.28970      -0.30620
C         1.63850       0.96260       0.24380
H         1.86700       1.14200       1.29440
C         2.01350      -0.49850      -0.17610
C         1.93790      -1.56040       0.86540
O         0.94430      -2.26100       1.06140
N         3.03270      -1.69070       1.63580
C         4.25730      -0.98200       1.53070
O         5.12910      -1.18620       2.33010
N         4.32640      -0.08200       0.51230
C         3.39960       0.04040      -0.58240
H         3.82140      -0.33800      -1.51620
H         5.23980       0.33990       0.41680
H         3.01620      -2.37900       2.37990
H         1.43680      -0.80320      -1.05150
H         0.60830       1.91340      -3.20550
H        -0.13790       1.46500       1.95330
O        -1.24540       0.01580       0.98900
H        -1.75530       3.07340       0.21910
H        -3.30670       1.29190      -0.20630
```

References:

1. Allen, S. J.; Giles, K.; Gilbert, T.; Bush, M. F. Ion mobility mass spectrometry of peptide, protein, and protein complex ions using a radio-frequency confining drift cell. *Analyst*, **2016**, *141*, 884-891. https://doi.org/10.1039/C5AN02107C.
2. Revercomb, H. E.; Mason, E. A. Theory of plasma chromatography/gaseous electrophoresis. Review. *Anal. Chem.* **1975**, *47*, 970–983. https://doi.org/10.1021/ac60357a043.
3. Zanotto, L. *et al.* High performance collision cross section calculation—HPCCS. *J. Comput. Chem.* **2018**, *39*, 1675–1681. https://doi.org/10.1002/jcc.25199.
